# Global research trends and clinical trial progress in varicose vein treatment: A decade of advancements (2014–2024)

**DOI:** 10.1097/MD.0000000000047592

**Published:** 2026-02-06

**Authors:** Yu Mao, Guangji Chen, Shiwei Zhou

**Affiliations:** a Department of Thyroid Surgery, The Affiliated Cancer Hospital of Xiangya School of Medicine, Central South University/Hunan Cancer Hospital, Changsha, Hunan, PR China; b Department of Thyroid Surgery, The Second Xiangya Hospital, Central South University, Changsha, Hunan, PR China; c Department of Surgery, University Hospital, Central South University, Changsha, Hunan, PR China.

**Keywords:** bibliometric analysis, endovenous treatment, patient-reported outcomes, research trends, varicose veins, vascular surgery

## Abstract

**Objective:**

To comprehensively examine global research developments in varicose vein treatment over the past decade by combining bibliometric analysis with an overview of clinical trial trends, highlighting shifts in therapeutic strategies and emerging priorities.

**Methods:**

A bibliometric assessment was carried out using the Web of Science Core Collection, focusing on English articles and reviews published between January 1, 2014 and December 31, 2024. Tools such as VOSviewer, CiteSpace, and SciExplorer were used to analyze publication patterns, geographic and institutional contributions, author collaborations, co-citation networks, keyword co-occurrences, and emerging research themes. Additionally, a comprehensive review of 374 clinical trials from PubMed was performed to explore trends in study design, treatment modalities, and patient-reported outcomes across the same period.

**Results:**

A total of 3110 publications were identified, with the annual output showing substantial growth, peaking in 2021. The leading contributors were the United States, China, and the United Kingdom, with prominent institutions like Imperial College London and Mayo Clinic being notable contributors. The analysis of key journals revealed a strong presence of Phlebology and The Journal of Vascular Surgery. Keyword analysis highlighted ongoing interest in “endovenous laser ablation” and “compression therapy,” while emerging topics such as “pelvic congestion syndrome,” “cyanoacrylate closure,” and “patient-reported outcomes” are gaining attention. The clinical trial review showed a steady rise in studies, particularly between 2014 and 2024, with a notable peak around 2015. Randomized controlled trials dominated, focusing on minimally invasive techniques such as endovenous laser ablation, RFA, and tissue adhesives. Geographic distribution aligned with the bibliometric analysis, with dominant contributions from the United States, China, and Europe. The primary focus of these trials included efficacy, safety, recurrence, and patient-centered outcomes, with a growing emphasis on long-term effects and the use of innovative devices.

**Conclusion:**

Over the past decade, research on varicose vein treatment has seen substantial progress, driven by developments in minimally invasive technologies and a patient-focused approach. The combined bibliometric and clinical trial analyses highlight a continuing trend toward personalized, tech-enabled therapies. Integrating these insights will be crucial for guiding future research towards sustained efficacy and individualized care strategies.

## 1. Introduction

Varicose veins, a manifestation of chronic venous insufficiency, are a common vascular disorder affecting millions of people worldwide.^[1,2]^ The condition is characterized by dilated, tortuous superficial veins, most frequently occurring in the lower extremities.^[3]^ Its prevalence increases with age and is more common among females. Although varicose veins are sometimes perceived as a cosmetic issue, they can cause significant symptoms and lead to serious complications if left untreated.^[4]^ Consequently, timely and effective management is essential in both primary care and specialized vascular practices.^[5,6]^

Over the past decade, treatment strategies for varicose veins have evolved considerably. Traditional surgical techniques, such as high ligation and stripping, have increasingly been replaced by minimally invasive modalities, including endovenous laser ablation (EVLA), radiofrequency ablation (RFA), foam sclerotherapy, and mechanochemical ablation.^[7]^ These newer techniques offer reduced recovery time, improved patient satisfaction, and comparable long-term efficacy.^[8-10]^ Alongside these advancements, the field has witnessed a surge in research output focusing on optimizing treatment efficacy, safety, patient-reported outcomes, and cost-effectiveness.^[11]^

Despite the growing body of literature, a comprehensive understanding of the knowledge structure and emerging trends in varicose vein treatment remains lacking.^[12,13]^ Bibliometric analysis offers a quantitative approach to mapping the development of scientific knowledge in a given field.^[14,15]^ By analyzing publication trends, authorship networks, institutional collaborations, keyword co-occurrences, and citation patterns, bibliometric studies provide valuable insights into the evolution, focus areas, and future directions of research.^[16]^

This study aims to conduct a comprehensive bibliometric analysis of the global research landscape in varicose vein treatment over the past decade (2014–2024). Utilizing advanced tools such as VOSviewer, CiteSpace, and SciExplorer, we seek to identify the most influential publications, authors, and institutions, as well as to detect research hotspots and emerging frontiers. The findings of this study are intended to guide clinicians, researchers, and policymakers in understanding the trajectory of scientific inquiry and fostering more targeted, collaborative, and impactful research in the management of varicose veins.

## 2. Materials and methods

### 2.1. Search strategy

**Figure 1.**
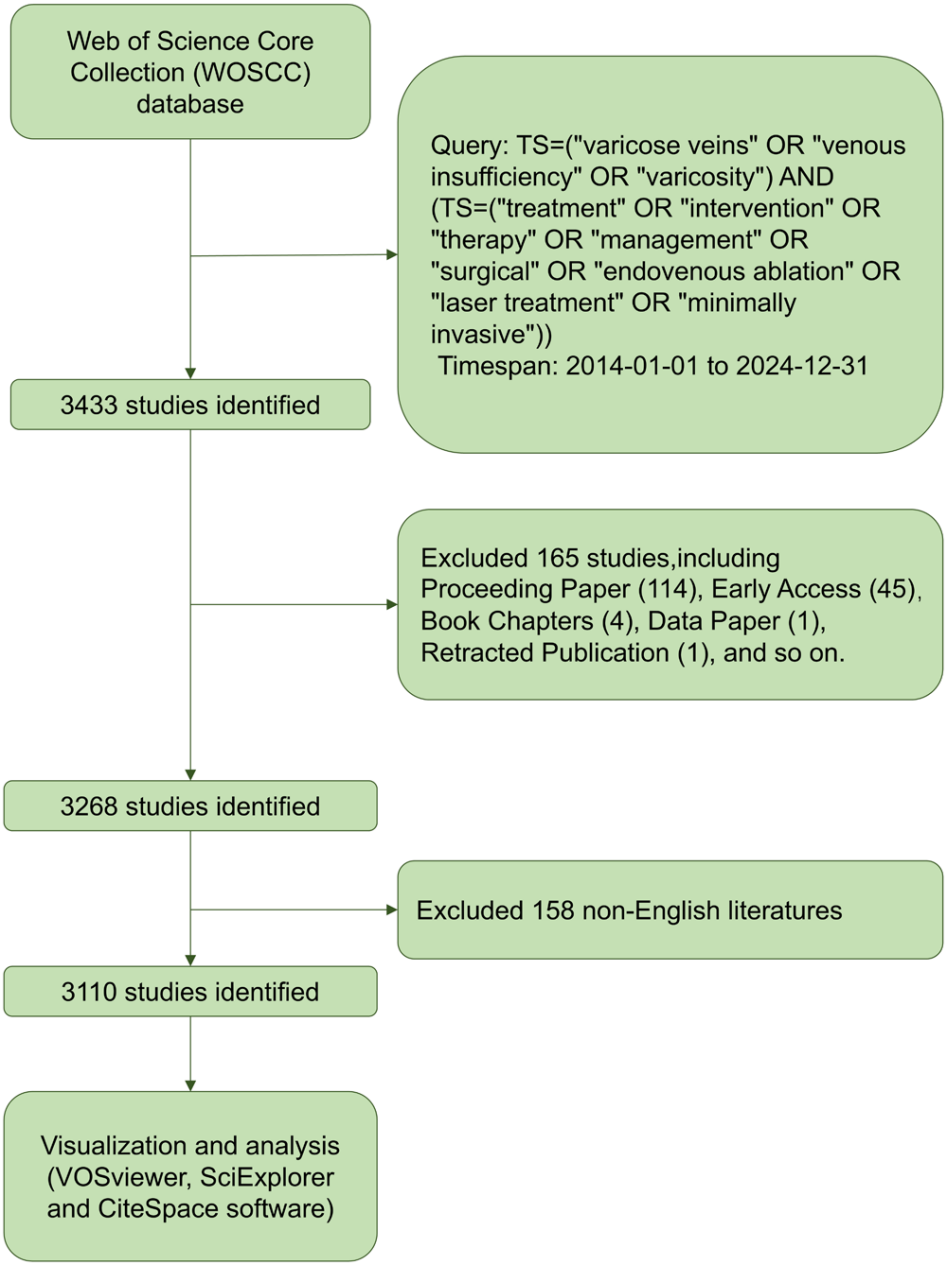
Flowchart of the literature search and study selection process for varicose vein treatment. Schematic representation outlining the systematic steps undertaken for literature identification, screening, eligibility assessment, and final inclusion of studies relevant to varicose vein treatment. OR = odds ratio, WoSCC = Web of Science Core Collection.

A comprehensive and systematic literature review was conducted to identify studies published between January 1, 2014 and December 31, 2024, focusing on the treatment of varicose veins. All publications were sourced from the Web of Science Core Collection database, and all publications were in English. The search query was: TS=(“varicose veins” OR “venous insufficiency” OR “varicosity”) AND (TS=(“treatment” OR “intervention” OR “therapy” OR “management” OR “surgical” OR “endovenous ablation” OR “laser treatment” OR “minimally invasive”)). Only research papers (articles) and reviews were included. The literature search and download tasks were independently carried out by Mao and Zhou, with any discrepancies resolved through discussion. We conducted the search and export operation on May 10, 2025, and compiled the final dataset in TEXT format. An overview of the data collection and analysis process is presented in Figure 1.

### 2.2. Data analysis

This study conducted a comprehensive bibliometric analysis using advanced visualization tools such as VOSviewer (version 1.6.19), CiteSpace (version 6.2.R4), and SciExplorer.^[17,18]^ These tools were specifically chosen for their ability to analyze and visualize the collected publications, providing valuable insights into the research landscape of varicose vein treatment. VOSviewer was particularly useful for constructing and visualizing bibliometric networks, illustrating the relationships between key research entities such as authors, institutions, journals, and countries, and generating clear, interpretable visualizations.^[19]^ CiteSpace was used to analyze research trends by detecting burst events and clustering citation patterns, helping to identify emerging research hotspots and trends in the field.^[20]^ SciExplorer was employed to analyze the evolution of research topics within the literature, offering further insights into the development of the field. By integrating these tools, this study constructed collaboration network maps, performed keyword co-occurrence analysis, and revealed key research dynamics in the field.

### 2.3. Analysis of clinical trials

In addition, a targeted review of clinical trials was performed to analyze the trends in experimental design, treatment modalities, and patient-centered outcomes. Clinical trial publications (identified via a dedicated search in PubMed) were extracted, and their annual publication counts were analyzed to assess temporal trends. Key trial characteristics, such as study type (e.g., randomized controlled trials), intervention methods (e.g., EVLA, RFA, cyanoacrylate closure), and primary endpoints (e.g., efficacy, safety, quality of life), were summarized. This supplemental analysis provided insight into the translation of research into clinical practice over the study period.

## 3. Results

### 3.1. Publication trends

**Figure 2.**
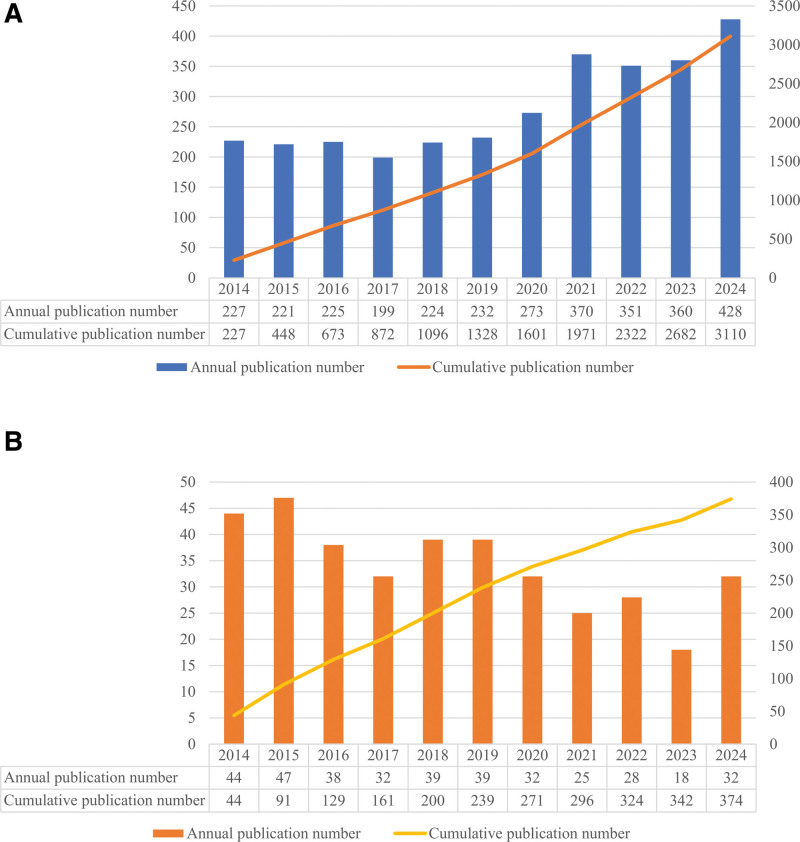
Trends in publications on varicose vein treatment over time. Annual publication counts depicting the progressive increase in (A) total research output, and (B) clinical trials within the field, highlighting growing scientific interest and activity.

A total of 3110 publications were identified, showing a steady upward trend in research on varicose vein treatment over the past decade, with the highest number of publications in 2021. The full trend is shown in Figure 2A.

The number of clinical trials conducted annually remained relatively stable over the past decade, with slight fluctuations – from 44 in 2014 to a peak of 47 in 2015, and a low point of 18 in 2023 (Fig. 2B). Despite some downward fluctuations, the overall trend indicates consistent clinical research activity, supporting ongoing innovation in varicose vein treatment (Table S1, Supplemental Digital Content, https://links.lww.com/MD/R338).

### 3.2. Geographical and institutional distribution

**Figure 3.**
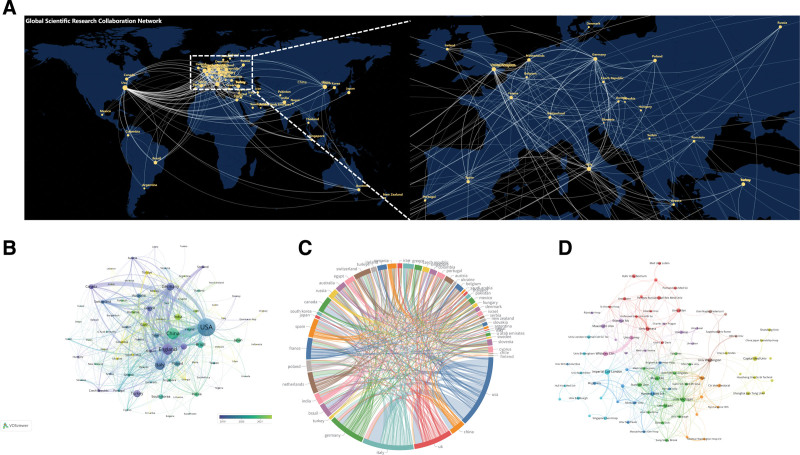
Geographic collaboration and publication metrics in varicose vein treatment research. (A) Global collaboration network map illustrating inter-country research partnerships; key countries are labeled, with yellow lines indicating collaborative links. (B) Bar chart showing recent publication volumes by country, identifying leading contributors. (C) Chord diagram visualizing the frequency and intensity of bilateral collaborations among prominent countries. (D) Institutional analysis chart presenting publication outputs over recent years, emphasizing principal research centers driving the field forward.

Research on varicose vein treatment is globally distributed, with major contributions from the United States, China, and the United Kingdom. As shown in Table 1, the United States accounts for 19.1% of total publications, followed by China (8.59%) and the United Kingdom (7.64%). Figure 3A–C illustrates the geographical distribution of both publications and citations. The United States reported 745 publications and 12,404 citations, with China and the United Kingdom also showing high numbers in both metrics.

**Table 1:** Top 10 countries and institutions with the most documents on research of varicose vein treatment.

Rank	Country	Centrality	Count (%)	Institution	Centrality	Count (%)
1	United States	0.39	745 (19.1%)	Imperial College London	0.12	46 (1.19%)
2	China	0.05	335 (8.59%)	University of Michigan	0.10	38 (0.98%)
3	The United Kingdom	0.14	298 (7.64%)	Whiteley Clinic	0.10	37 (0.96%)
4	Italy	0.21	272 (6.97%)	Mayo Clinic	0.04	34 (0.88%)
5	Germany	0.11	172 (4.41%)	Capital Medical University	0.01	33 (0.85%)
6	Turkey	0.04	148 (3.79%)	Harvard Medical School	0.14	32 (0.83%)
7	Brazil	0.02	128 (3.28%)	University of Miami	0.14	30 (0.78%)
8	India	0.08	121 (3.10%)	Erasmus University Rotterdam	0.02	29 (0.75%)
9	Netherlands	0.05	111 (2.85%)	New York University	0.02	29 (0.75%)
10	Poland	0.03	103 (2.64%)	University of Washington	0.01	29 (0.75%)

At the institutional level, Table 1 and Figure 3D display the leading research institutions in this field. Imperial College London recorded the highest number of citations (828), followed by Harvard Medical School and Mayo Clinic. Other institutions with notable contributions include McMaster University, Erasmus University, and Whiteley Clinic.

### 3.3. Journals and co-cited journals

**Figure 4.**
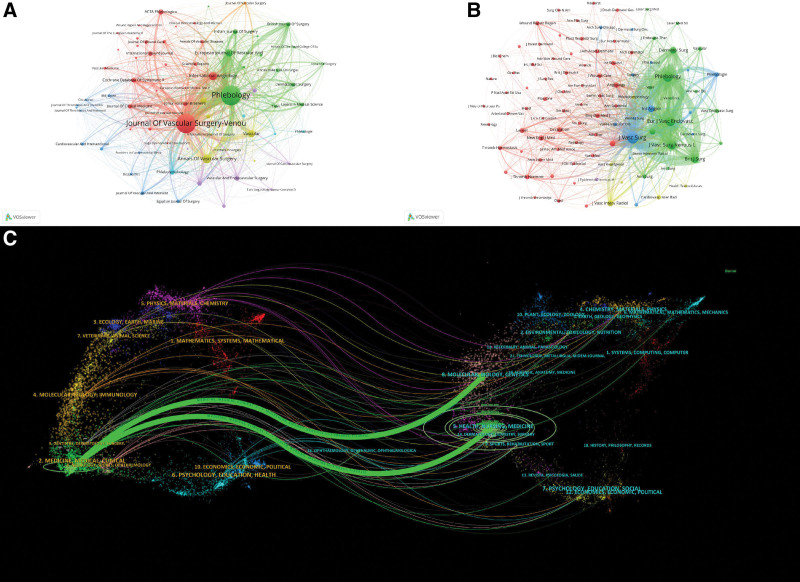
Journal contribution and citation network analysis in varicose vein treatment. (A) Visualization of leading journals publishing varicose vein treatment research. (B) Co-citation network map revealing relationships and citation patterns between journals. (C) Dual-map overlay displaying citation trajectories between citing and cited journals, highlighting interdisciplinary connections.

Research on varicose vein treatment is published across a wide range of journals. As shown in Figure 4A and Table 2, *Phlebology* leads with 379 publications (12.19%) and an impact factor (IF) of 1.6 (Q3), followed by *Journal of Vascular Surgery: Venous and Lymphatic Disorders* with 359 publications (11.54%) and an IF of 2.8 (Q2). Other journals with notable publication volume include *Annals of Vascular Surgery* (85 publications, IF: 1.4, Q3) and *International Angiology* (77 publications, IF: 1.5, Q3).

**Table 2:** The top 10 productive journals related to varicose vein treatment.

Rank	Journal	Publications (%)	IF (JCR2023)	JCR quartile
1	Phlebology	379 (12.19%)	1.6	Q3
2	Journal of Vascular Surgery: Venous and Lymphatic Disorders	359 (11.54%)	2.8	Q2
3	Annals of Vascular Surgery	85 (2.73%)	1.4	Q3
4	International Angiology	77 (2.48%)	1.5	Q3
5	Vascular	63 (2.03%)	1.0	Q4
6	European Journal of Vascular and Endovascular Surgery	60 (1.93%)	5.7	Q1
7	Cureus Journal of Medical Science	45 (1.45%)	1.1	Q3
8	Acta Phlebologica	34 (1.09%)	0.4	Q4
9	Phlebolymphology	32 (1.03%)	0.2	Q4
10	Vascular and Endovascular Surgery	31 (1.00%)	1.4	Q3

IF = impact factor, JCR = journal citation reports.

Co-citation analysis based on citation frequency is shown in Figure 4B and Table 3. *Journal of Vascular Surgery* ranks highest with 5888 citations, followed by *Phlebology* (4153 citations), *European Journal of Vascular and Endovascular Surgery* (2473 citations), and *Journal of Vascular Surgery: Venous and Lymphatic Disorders* (2038 citations). Other journals such as *British Journal of Surgery* (IF: 8.7, Q1) and *Cochrane Database of Systematic Reviews* (IF: 8.8, Q1) also exhibit high citation counts and impact factors.

**Table 3:** Top 10 journals for co-citation of varicose vein treatment.

Rank	Cited journal	Citation	IF (JCR2023)	JCR quartile
1	Journal of Vascular Surgery	5888	3.9	Q1
2	Phlebology	4153	1.6	Q3
3	European Journal of Vascular and Endovascular Surgery	2473	5.7	Q1
4	Journal of Vascular Surgery: Venous and Lymphatic Disorders	2038	2.8	Q2
5	British Journal of Surgery	1754	8.7	Q1
6	International Angiology	1501	1.5	Q3
7	Dermatologic Surgery	1293	2.5	Q1
8	Annals of Vascular Surgery	1261	1.4	Q3
9	Journal of Vascular and Interventional Radiology	943	2.6	Q2
10	Cochrane Database of Systematic Reviews	868	8.8	Q1

IF = impact factor, JCR = journal citation reports.

Figure 4C presents a dual-overlay map that illustrates co-citation relationships in the field. The overlay highlights citation flows between disciplines such as “Medicine, Medical, Clinical” and “Molecular Biology, Immunology.”

### 3.4. Authors and co-cited authors

**Figure 5.**
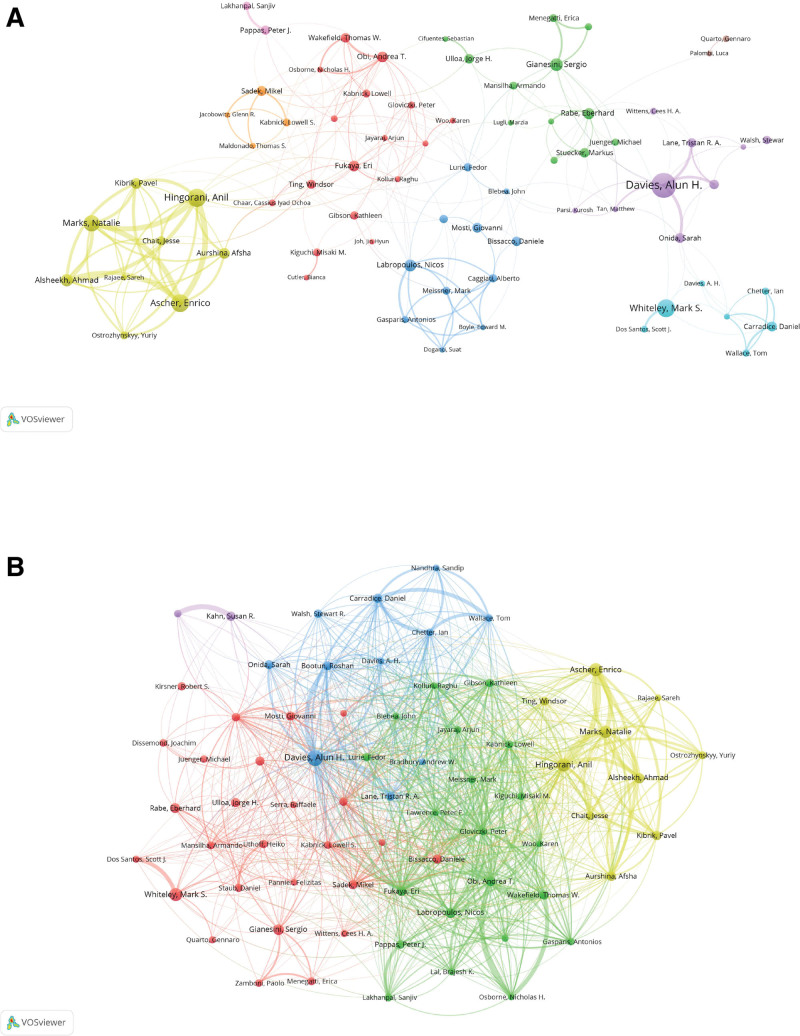
Author productivity and co-citation patterns in varicose vein treatment research. (A) Network map of the most prolific authors contributing to the literature. (B) Co-citation map identifying influential authors whose works significantly shape the field.

Figure 5A illustrates the author network in varicose vein treatment. Alun H. Davies is identified as the most prolific author, with 44 publications and 811 citations. He is followed by Anil Hingorani (31 publications, 292 citations) and Mark S. Whiteley (30 publications, 412 citations). Joseph D. Raffetto is noted for having 13 publications and 1035 citations.

Figure 5B presents the co-cited author analysis. Peter Gloviczki received 759 citations, followed by Eberhard Rabe (663 citations) and Nicos Labropoulos (522 citations). Alun H. Davies is also among the frequently co-cited authors. Table 4 lists the top authors and co-cited authors based on their publication counts and citation metrics.

**Table 4:** Top 10 authors and co-cited authors on research of varicose vein treatment.

Rank	Authors	Location	Count	Co-cited authors	Location	Citations
1	Alun H Davies	The United Kingdom	44	Peter Gloviczki	United States	759
2	Anil Hingorani	United States	31	Eberhard Rabe	Germany	663
3	Enrico Ascher	United States	30	Nicos Labropoulos	United States	522
4	Mark S Whiteley	The United Kingdom	30	Susan R Kahn	Canada	514
5	Natalie Marks	United States	27	Thomas M Proebstle	Netherlands	499
6	Sergio Gianesini	Italy	21	Seshadri Raju	United States	487
7	Ahmad Alsheekh	United States	20	Fedor Lurie	United States	456
8	Nicos Labropoulos	United States	19	Jose I Almeida	United States	377
9	Eberhard Rabe	Germany	18	Lars H Rasmussen	Denmark	372
10	Afsha Aurshina	United States	17	Bo Eklöf	United States	346

### 3.5. Co-cited references and citation bursts

**Figure 6.**
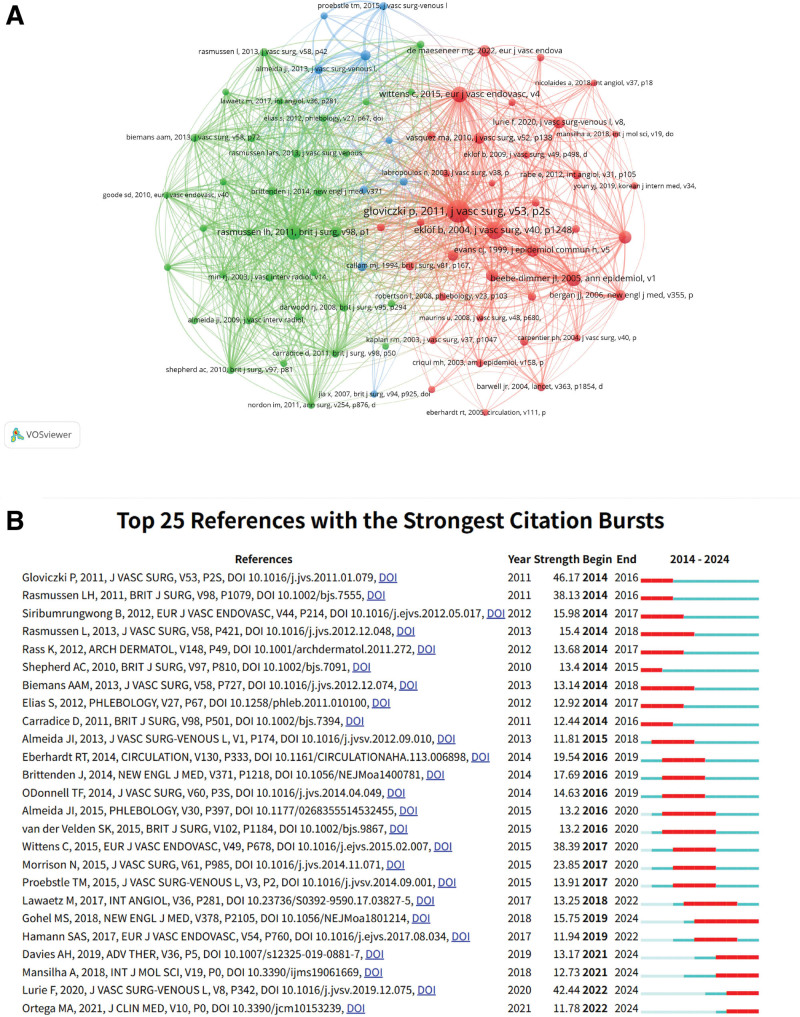
Reference citation analysis in varicose vein treatment. (A) Visualization of the most frequently co-cited references. (B) Timeline of the top 25 references exhibiting strongest citation bursts, indicating seminal contributions and emerging influential works over time.

Figure 6A illustrates the co-cited reference network in the field of varicose vein treatment. The reference by Gloviczki, P (2011) appears most frequently, with multiple interconnections to other studies. Other highly co-cited references include Rasmussen, LH (2013) and Eklöf, B (2004), which address clinical, etiological, anatomical, and pathophysiological (CEAP) classification and chronic venous diseases. The network includes color coding to indicate citation bursts over time.

Figure 6B and Table 5 present the top 25 references with the strongest citation bursts. Gloviczki’s (2011) reference ranks first with 531 citations, followed by Rasmussen’s (2011) randomized clinical trial and Wittens’ (2015) guidelines. Lurie’s (2020) update to the CEAP classification system is also included. Table 5 summarizes the top 10 co-cited references, including publications by Gloviczki (2011) and Eklöf (2004).

**Table 5:** Ranking of the top 10 co-cited references for varicose vein treatment.

Rank	Reference	Citations	Year	First author	Journal
1	The care of patients with varicose veins and associated chronic venous diseases: clinical practice guidelines of the Society for Vascular Surgery and the American Venous Forum	531	2011	Peter Gloviczki	Journal of Vascular Surgery
2	Revision of the CEAP classification for chronic venous disorders: consensus statement	333	2004	Bo Eklöf	Journal of Vascular Surgery
3	Editor’s Choice – Management of Chronic Venous Disease: Clinical Practice Guidelines of the European Society for Vascular Surgery (ESVS)	277	2015	C Wittens	European Journal of Vascular and Endovascular Surgery
4	Randomized clinical trial comparing endovenous laser ablation, radiofrequency ablation, foam sclerotherapy and surgical stripping for great saphenous varicose veins	250	2011	LH Rasmussen	British Journal of Surgery
5	The epidemiology of chronic venous insufficiency and varicose veins	248	2005	Jennifer L Beebe-Dimmer	Annals of Epidemiology
6	Prevalence of varicose veins and chronic venous insufficiency in men and women in the general population: Edinburgh Vein Study	186	1999	CJ Evans	Journal of Epidemiology & Community Health
7	Chronic venous insufficiency	185	2014	Robert T Eberhardt	Circulation
8	The 2020 update of the CEAP classification system and reporting standards	169	2020	Fedor Lurie	Journal of Vascular Surgery: Venous and Lymphatic Disorders
9	Management of venous leg ulcers: clinical practice guidelines of the Society for Vascular Surgery^®^ and the American Venous Forum	166	2014	Thomas F O’Donnell Jr	Journal of Vascular Surgery
10	Editor’s Choice – European Society for Vascular Surgery (ESVS) 2022 Clinical Practice Guidelines on the Management of Chronic Venous Disease of the Lower Limbs	161	2022	Marianne G De Maeseneer	European Journal of Vascular and Endovascular Surgery

CEAP = clinical, etiological, anatomical, and pathophysiological, ESVS = European Society for Vascular Surgery.

### 3.6. Hotspots and frontiers

**Figure 7.**
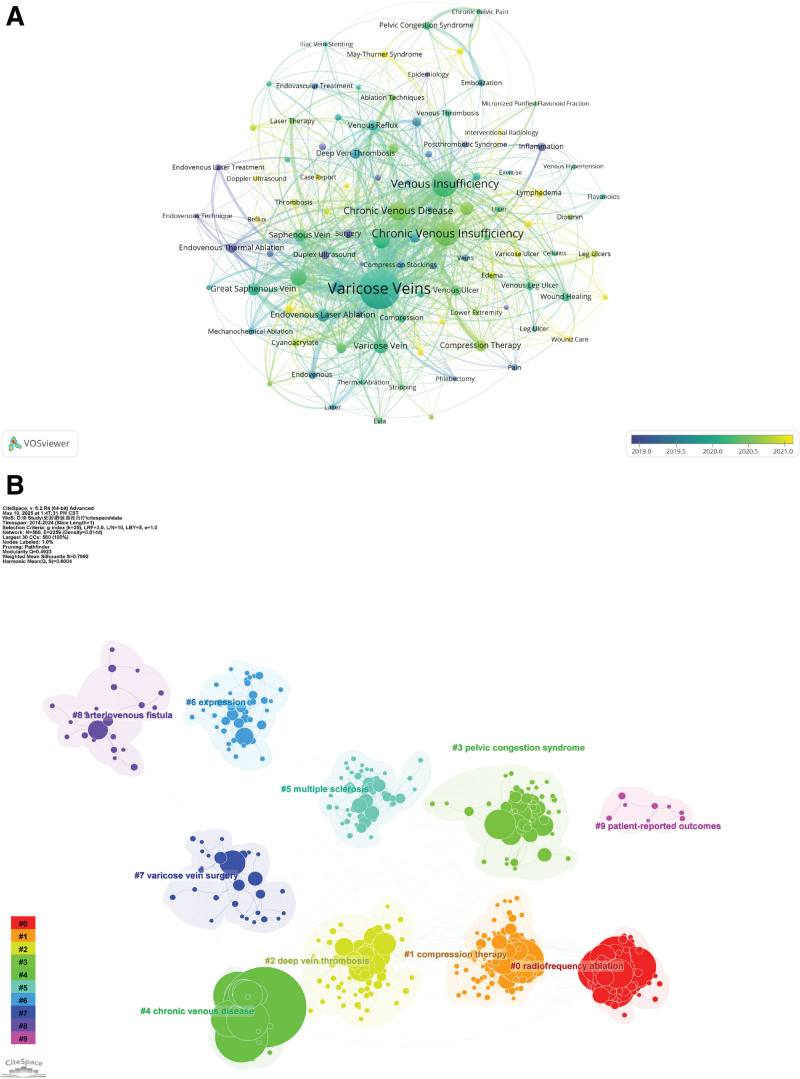
Keyword dynamics and thematic clustering in varicose vein treatment research. (A) Temporal keyword map demonstrating evolution and shifts in research focus. (B) Cluster analysis of related keywords uncovering principal thematic areas and research topics within the domain.

Figure 7A presents a co-occurrence network of keywords in varicose vein treatment research. Frequently appearing terms include “Varicose Veins,” “Chronic Venous Insufficiency,” and “radiofrequency ablation.” These terms are located at the center of several interconnected clusters. Other central keywords include “Saphenous Vein” and “endovenous laser ablation.” Additional terms such as “Pelvic Congestion Syndrome” and “Patient-Reported Outcomes” also appear within the network.

Figure 7B highlights the major thematic clusters. Keywords such as “Chronic Venous Disease” and “Deep Vein Thrombosis” are grouped into distinct clusters. Other clustered terms include “Compression Therapy” and “Endovenous Ablation.” The modularity (*Q*) value of 0.751 indicates a well-structured network with clearly separated thematic clusters. The silhouette (*S*) value of 0.87 suggests high internal consistency within each cluster.

## 4. Discussion

Over the past decade, research on varicose vein treatment has shown a notable and steady increase in academic output. The annual publication count nearly doubled from 227 in 2014 to 428 in 2024. A significant peak in 2021 coincided with the COVID-19 pandemic, which likely intensified global attention on chronic venous disorders, outpatient safety, and procedural efficiency amid healthcare system strain. The temporary decline in 2022 likely reflects a post-pandemic reallocation of research priorities, with investigators resuming pre-pandemic agendas and funding shifting back to non-COVID topics.^[21]^ Nonetheless, the overall trajectory has remained positive. The steep rise in cumulative publications from 800 in 2017 to over 2300 in 2022 suggests that varicose vein treatment has matured from a niche focus into a widely recognized area of vascular research.

Geographical and institutional analyses revealed that the United States maintains a dominant position in both output and citation impact, contributing nearly one-fifth of all publications and receiving over 12,000 citations. China and the United Kingdom follow in overall productivity, though differences in average citation rates suggest disparities in international academic influence. This discrepancy may stem from varying levels of research funding, language accessibility, and cross-border collaboration. On the institutional level, major academic centers such as Imperial College London, Harvard Medical School, and Mayo Clinic demonstrated strong presence in both productivity and impact, indicating a sustained commitment to vascular research from high-volume and research-intensive environments. The appearance of nonacademic clinics like Whiteley Clinic also indicates the important role that specialized private centers can play in shaping clinical innovation and knowledge dissemination, highlighting the intersection between academic inquiry and real-world procedural practice.

In terms of journal distribution, the publication landscape is anchored by specialized vascular journals such as *Phlebology* and the *Journal of Vascular Surgery: Venous and Lymphatic Disorders*, which accounted for the highest article counts. These journals provide a focused platform for the exchange of procedural innovations, clinical outcomes, and disease classification updates. Meanwhile, high-impact journals like the *British Journal of Surgery* and the *Cochrane Database of Systematic Reviews*, though publishing fewer varicose vein articles, occupy central positions in co-citation networks, underscoring their broader influence and perceived methodological rigor. This pattern demonstrates that while field-specific outlets drive volume, cross-disciplinary journals amplify reach and credibility.^[22]^

The author and co-citation networks further highlighted the structure of academic leadership in this area. Alun H. Davies stands out as the most prolific and co-cited author, followed by Anil Hingorani and Mark S. Whiteley in terms of publication count, and Joseph Raffetto and Peter Gloviczki in terms of citation strength. These individuals represent a relatively small, yet highly interconnected group of experts whose work has strongly influenced both clinical guidelines and procedural advancements. Their prominence suggests a core academic cohort that not only produces primary research but also shapes the conceptual framework and best practices adopted across the field.

Analysis of co-cited references and citation bursts confirmed the continued influence of landmark publications. Foundational guidelines such as Gloviczki et al (2011), Rasmussen (2011), and Wittens (2015) remain at the center of reference networks, suggesting that much of the field still builds upon these seminal works. The persistence of strong citation bursts for clinical recommendations, particularly those involving CEAP classification and treatment algorithms, reflects the field’s reliance on standardized frameworks for both diagnosis and procedural decision-making. Additionally, the inclusion of recent updates such as Lurie (2020) among high-burst references indicates ongoing efforts to refine these systems in light of new evidence and techniques. This ongoing citation activity reflects not only academic relevance but also clinical utility.

The keyword co-occurrence and clustering analyses revealed several persistent research hotspots in the treatment of varicose veins. Dominant terms such as “endovenous laser ablation,” “radiofrequency ablation,” and “compression therapy” formed the core of multiple clusters, indicating that these treatment modalities have remained at the forefront of scholarly attention. This aligns with the widespread clinical shift over the past decade toward minimally invasive techniques that offer advantages in procedural safety, patient comfort, and cosmetic outcomes compared to traditional surgical approaches such as high ligation and stripping.^[23,24]^ These methods have become the standard of care in many countries and are increasingly supported by international guidelines, contributing to their prominence in both clinical and academic settings.^[25]^

The central positioning of terms like “saphenous vein,” “chronic venous insufficiency,” and “venous reflux” underscores the foundational importance of understanding venous hemodynamics in treatment selection and outcome evaluation.^[26]^ These terms serve not only as frequently studied entities but also as conceptual anchors across thematic clusters, reflecting their cross-cutting relevance to both pathophysiological exploration and procedural innovation. Their repeated appearance across disparate clusters suggests that while the techniques evolve, the biological and anatomical substrates of disease remain constant focal points, driving ongoing refinements in diagnostics, procedural indications, and long-term surveillance strategies.^[27]^

Beyond these well-established topics, the analysis identified a range of emerging research frontiers that signal evolving priorities and expanding awareness in the field. One notable area is the increasing academic attention to “pelvic congestion syndrome,” which has historically been under-recognized and misdiagnosed.^[28]^ While often excluded from traditional varicose vein frameworks, pelvic congestion syndrome is now gaining traction as a clinically significant venous disorder, particularly in premenopausal women presenting with chronic pelvic pain.^[29,30]^ The inclusion of pelvic congestion syndrome in recent bibliometric clusters suggests that researchers are expanding the scope of chronic venous disease beyond the lower limbs, acknowledging the broader systemic manifestations of venous insufficiency.^[31,32]^ As awareness increases, so too does the demand for improved diagnostic tools, imaging modalities (e.g., transvaginal Doppler, MRI venography), and interventional approaches (e.g., ovarian vein embolization), which may become focal points in future studies.^[33-35]^

Another prominent trend is the rise of patient-centered outcomes, as evidenced by the co-occurrence and burst strength of keywords such as “patient-reported outcomes,” “quality of life,” and “satisfaction.” This reflects a paradigmatic shift in how procedural success is defined – from technical endpoints like occlusion rates and recurrence to more holistic assessments encompassing physical symptoms, psychosocial wellbeing, return to activity, and treatment experience. The growing incorporation of validated instruments such as the chronic venous insufficiency questionnaire and VEINES-QOL/Sym questionnaires in clinical trials and cohort studies further illustrates this trend.^[36]^ This movement toward value-based care is particularly relevant in varicose vein treatment, where interventions are elective and patient expectations often extend beyond clinical resolution to aesthetic and functional recovery.

The evolution of procedural techniques is also evident in the emergence of keywords related to novel therapies, including “cyanoacrylate closure,” “mechanochemical ablation,” and “foam sclerotherapy.” These techniques are gaining attention for their simplified delivery, shorter procedure times, and reduced need for post-procedural compression.^[37,38]^ While long-term data are still being collected, their increasing prevalence in the literature suggests growing academic and commercial interest. In particular, cyanoacrylate-based closure systems have been the focus of multiple multi-center studies and regulatory approvals, indicating a trajectory toward broader adoption and potential inclusion in future guideline updates.^[39,40]^ These trends highlight the dynamic nature of the therapeutic landscape, in which innovation is driven not only by efficacy, but also by workflow efficiency, patient tolerance, and economic feasibility.

The high modularity (*Q* = 0.751) and silhouette (*S* = 0.87) values observed in the clustering analysis support the interpretation that research on varicose vein treatment has become increasingly stratified into distinct thematic domains. These include clinical management, imaging and diagnostics, device innovation, pathophysiology, and patient experience. The well-defined structure of these clusters suggests that while research depth within each domain is increasing, integration across domains remains a challenge. For example, few studies simultaneously address biomechanical factors, imaging biomarkers, and long-term quality-of-life metrics within the same cohort. This gap points to the need for more interdisciplinary research that bridges vascular biology, medical technology, behavioral science, and health economics.

Additionally, the growing use of machine learning, image-based AI interpretation, and personalized risk modeling – though still underrepresented in this bibliometric sample – may soon contribute to a new frontier in personalized venous care. As digital tools become more widely implemented, future research may shift from generalized outcome prediction to individualized treatment planning, particularly in complex or recurrent disease presentations.^[41,42]^

Collectively, these findings suggest that varicose vein research is undergoing both vertical deepening – through more precise diagnostic tools, advanced technologies, and refined pathophysiological models – and horizontal broadening – through the inclusion of patient-centered, psychosocial, and systemic considerations. As the field continues to evolve, future studies that integrate clinical innovation with real-world outcomes and societal value are likely to drive the next generation of guidelines and care models.

A major strength of this study lies in its robust bibliometric methodology, which incorporated data from a 10-year span and utilized multiple analytical platforms, including VOSviewer, CiteSpace, and SciExplorer. This allowed for detailed mapping of authorship networks, keyword evolution, and citation dynamics. Additionally, the study provides a balanced view of both established research hotspots and emerging frontiers, offering a comprehensive perspective on the field’s development.

However, certain limitations must be acknowledged. First, the analysis was limited to English-language publications indexed in the Web of Science Core Collection, which may have excluded relevant studies published in other languages or databases. Second, bibliometric indicators such as citation count and co-occurrence frequency may not fully reflect the clinical significance or methodological rigor of individual studies. Third, although keyword clustering reveals thematic relationships, it does not capture the nuance of study design or patient population characteristics. Future research should consider incorporating other databases (e.g., Scopus, Cochrane Library), full-text semantic analysis to enhance the granularity and generalizability of findings.

### 4.1. Clinical trials research progress in varicose vein treatments

**Figure 8.**
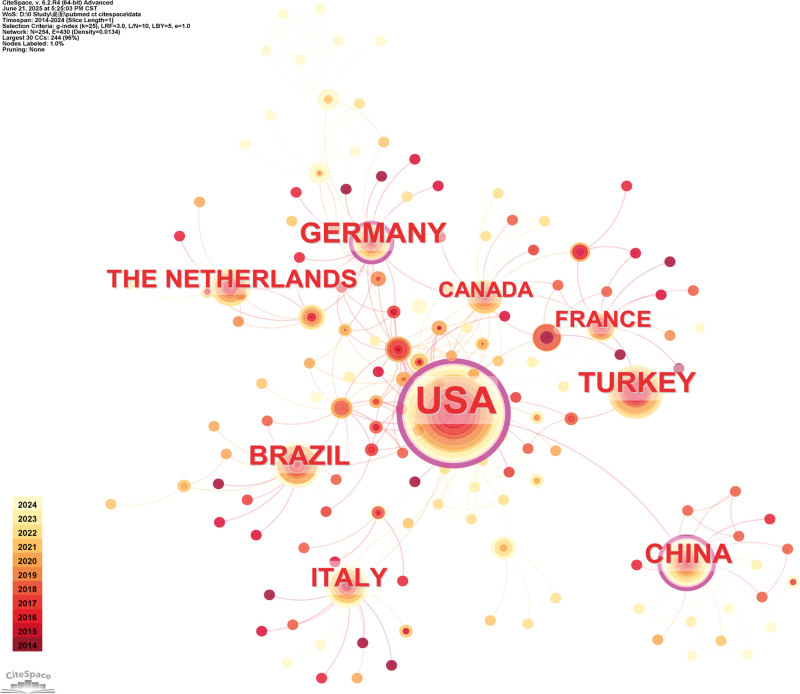
Visualization of international collaboration networks and publication metrics in clinical trials related to varicose vein treatment. Node size reflects the publication volume of each country/region in clinical trials on varicose vein treatment. Nodes with a significant central role are indicated by a purple outer circle, representing a betweenness centrality >0.1.

The geographic distribution analysis indicates that, among the countries with the highest publication volumes, the United States, China, and Germany exhibit betweenness centrality values exceeding 0.1 (Fig. 8). This highlights their pivotal roles in the global landscape of clinical trials for varicose vein treatment, reflecting their influence in research collaboration and knowledge dissemination. The prominent centrality of these countries underscores their leadership in driving innovation, standardization, and large-scale clinical studies, which are critical for establishing evidence-based practices. Furthermore, the increasing research activity in these regions signifies a growing commitment to improving treatment outcomes and fostering international cooperation in Varicose Vein Treatments.

The predominant therapeutic modalities examined include EVLA, RFA, and the emerging use of cyanoacrylate glue closure. Among these, EVLA remains the most extensively studied technique, supported by numerous randomized controlled trials, which account for over 70% of the examined trials, demonstrating high evidentiary standards. RFA has also been widely evaluated, often serving as a comparator in head-to-head trials. Notably, in recent years, the use of cyanoacrylate-based glue closure has experienced rapid growth, reflecting its potential as a minimally invasive alternative with promising safety and efficacy profiles.

The primary focus of these clinical investigations encompasses not only treatment efficacy and safety but also patient-centered outcomes such as quality-of-life, patient satisfaction, and long-term recurrence rates. This trend underscores a paradigm shift toward holistic assessment and personalized treatment strategies. Furthermore, emerging technologies, including adhesive closure techniques and image-guided interventions, are actively evaluated, indicating a future-oriented approach toward minimally invasive and individualized therapies.

Overall, clinical trials have significantly contributed to the evidence base guiding varicose vein treatment, facilitating comparative effectiveness research and informing evidence-based clinical practice. The continuous expansion of high-quality randomized studies and multi-center collaborations is expected to refine treatment algorithms, enhance long-term outcomes, and promote patient-centered care in this evolving therapeutic landscape.

## 5. Conclusion

This bibliometric analysis provides a comprehensive overview of global research in varicose vein treatment over the past decade. Publication volume steadily increased, with major contributions from the United States, China, and Germany, which also exhibited high centrality, highlighting their influential roles. The research shifted from traditional surgery toward minimally invasive, patient-centered therapies, with core focus on endovenous procedures, hemodynamics, and guidelines. Clinical trial data reveal sustained efforts in evidence generation, primarily through randomized controlled studies of EVLA, RFA, and new closure techniques, emphasizing a focus on safety, efficacy, and long-term outcomes. The field is becoming increasingly modular, with diverse themes evolving in parallel. Future progress depends on greater interdisciplinary integration and a focus on real-world, long-term results to guide clinical practice.

## Author contributions

**Data curation:** Yu Mao.

**Formal analysis:** Yu Mao.

**Methodology:** Guangji Chen.

**Software:** Guangji Chen.

**Supervision:** Shiwei Zhou.

**Writing – original draft:** Yu Mao.

**Writing – review & editing:** Guangji Chen, Shiwei Zhou.

## Supplementary Material


