# Supplementary material for: Global research trends and clinical trial progress in varicose vein treatment: A decade of advancements (2014–2024)
Source: Medicine (Baltimore). 2026 Feb 6;105(6):e47592. doi: 10.1097/MD.0000000000047592 (PMC12885685; doi:10.1097/MD.0000000000047592)
Supplement: Supplementary file 1 [file medi-105-e47592-s001.docx]

Supplementary table 1. List of the 374 clinical trials on varicose vein treatment.

| Published Year | Title | Journal | Author | DOI |
| --- | --- | --- | --- | --- |
| 2024 | EndoVenous-assisted invaginated stripping of the great saphenous vein: A pilot and feasibility study. | Phlebology | Alexiou, V. G., A. Vassiliou, M. Mitsis and M. Peroulis | 10.1177/02683555241257858 |
| 2024 | Femoral vein stenting versus endovenectomy as adjuncts to iliofemoral venous stenting in extensive chronic iliofemoral venous obstruction. | Phlebology | Alhewy, M. A., A. A. Abdelhafez, M. H. Metwally, E. A. E. Ghazala, A. M. Khedr, A. A. Khamis, H. Gado, W. A. A. Abd-Elgawad, A. El Sayed and A. A. Abdelmohsen | 10.1177/02683555241236824 |
| 2024 | Outcomes of Cyanoacrylate Closure Versus Radiofrequency Ablation for the Treatment of Incompetent Great Saphenous Veins. | Ann Vasc Surg | Alhewy, M. A., E. M. Abdo, E. A. E. Ghazala, A. A. Khamis, H. Gado, W. A. A. Abd-Elgawad, A. A. Abdelhafez, A. El Sayed, A. M. Khedr and H. A. M. Mosaed | 10.1016/j.avsg.2023.08.016 |
| 2024 | COmpressioN following endovenous TreatmenT of Incompetent varicose veins by sclerotherapy (CONFETTI). | J Vasc Surg Venous Lymphat Disord | Belramman, A., R. Bootun, T. R. A. Lane and A. H. Davies | 10.1016/j.jvsv.2023.101729 |
| 2024 | Single-session mechanical thrombectomy for iliofemoral deep vein thrombosis using a dual mechanism of action device combining basket and rotational thrombectomy. | J Vasc Surg Venous Lymphat Disord | Black, S. A., N. Thulasidasan, L. Benton, G. J. O'Sullivan, M. Konteva, I. S. Petrov, S. R. Walsh and M. Lichtenberg | 10.1016/j.jvsv.2024.101866 |
| 2024 | Detection and quantitation of venous leg edema with the edema ruler and ultrasound. | Phlebology | Calzon, M. E., J. Blebea and C. Pittman | 10.1177/02683555241258283 |
| 2024 | Is Kinesio taping acutely effective for peripheral tissue perfusion in women with mild to moderate chronic venous insufficiency? A randomized controlled trial. | J Bodyw Mov Ther | Carvalho, M. L. V., V. M. Caceres, I. O. Nascimento, H. S. Costa, P. H. S. Figueiredo, V. P. Lima, D. P. Monteiro and D. A. G. Pereira | 10.1016/j.jbmt.2024.04.017 |
| 2024 | High intensity focused ultrasound in treating great saphenous vein incompetence: Perioperative and 1-year outcomes. | Phlebology | Casoni, P., D. Bissacco, M. Pizzamiglio and E. Nanni | 10.1177/02683555241243161 |
| 2024 | Axial Ablation versus Terminal Interruption of the Reflux Source (AAVTIRS): A Randomised Controlled Trial. | Vasc Endovascular Surg | Cr, K., W. D, T. M, A. T, T. W and W. Sr | 10.1177/15385744241265750 |
| 2024 | Effects of lower limb muscle strengthening on interface pressure in older adults undergoing inelastic compression: Randomized controlled clinical trial. | Phlebology | Da Matta, E. S., G. Mosti, V. D. S. Corralo, G. P. Moura, L. B. Quadros and C. A. De Sá | 10.1177/02683555241235042 |
| 2024 | Safety of synchronous prophylactic ablation of the anterior saphenous vein in patients undergoing great saphenous vein thermal ablation- 6 months follow-up data of the SYNCHRONOUS study. | Phlebology | Dietrich, C. K., T. Hirsch, K. Hartmann, T. Mattausch, H. C. Wenzel, P. Zollmann, J. Veltman, T. K. Weiler, G. Lengfellner, L. Müller, M. Stücker, F. Pannier, L. Uhlmann and C. Müller-Christmann | 10.1177/02683555241257840 |
| 2024 | Compression therapy after endovenous laser ablation: Patient compliance and impact on clinical outcome. | Phlebology | Dietrich, C. K., M. Stucker, K. Hartmann, T. Hirsch, T. Mattausch, H. C. Wenzel, P. Zollmann, J. Veltman, T. K. Weiler, G. Lengfellner, L. Müller, F. Pannier, C. Cussigh, L. Uhlmann and C. Müller-Christmann | 10.1177/02683555241249222 |
| 2024 | Treatment of varicose great saphenous vein with endovenous laser alone or combined with eco-guided foam sclerotherapy: A randomized controlled trial. | Phlebology | Duarte, F., D. M. de Souza, A. Regueira Filho, L. J. Bazzanella, F. Del Castanhel and G. R. de Oliveira Filho | 10.1177/02683555241263224 |
| 2024 | Comparison of the effects of training in the standing and lying positions on the quality of life and clinical symptoms in women with mild varicose veins: A randomized controlled trial. | J Vasc Nurs | Ehteshami Puya, E., N. Khoshraftar Yazdi, S. A. A. Hashemi Javaheri, H. Taheri and A. Jafarzadeh Esfehani | 10.1016/j.jvn.2024.05.003 |
| 2024 | Effects of light compression on chronic venous disease, edema and comfort in women during pregnancy and postpartum period: a prospective randomized study. | Int Angiol | Frydrych-Szymonik, A., K. Ochałek, A. Jankowicz-Szymańska and Z. Szyguła | 10.23736/s0392-9590.24.05208-8 |
| 2024 | Challenging conventions: Reconsidering the indication for endovenous ablation in CEAP 2 patients. | Phlebology | Guven, H., T. Taner and M. S. Atasoy | 10.1177/02683555241260542 |
| 2024 | Varicose Vein Education and Informed coNsent (VVEIN) Study: A Randomized Controlled Pilot Feasibility Study. | Ann Vasc Surg | Kiernan, A., F. Boland, P. Naughton, D. Moneley, F. Doyle and D. W. Harkin | 10.1016/j.avsg.2024.02.030 |
| 2024 | Vitis Vinifera Seed Extract Versus Micronized Purified Flavonoid Fraction for Patients with Chronic Venous Disease: A Randomized Noninferiority Trial. | Ann Vasc Surg | Kim, S. M., J. H. Joh, I. M. Jung, M. J. Kim, S. S. Lee, H. P. Hwang, J. M. Kang, H. J. Jung, S. S. Yang, S. K. Min, Y. S. Yoo, J. G. Gwon, H. S. Park and T. Lee | 10.1016/j.avsg.2024.06.011 |
| 2024 | Ferumoxytol-enhanced MR venography for mapping lower-extremity venous networks and evaluating varicose veins in patients with diabetes. | Eur Radiol | Liu, Y., B. Cao, X. Wang, J. Zhong, Z. Li, R. Peng, D. Zhao, N. Gu and Q. Yang | 10.1007/s00330-024-10772-x |
| 2024 | Evaluating pharmacological THRomboprophylaxis in Individuals undergoing superficial endoVEnous treatment across NHS and private clinics in the UK: a multi-centre, assessor-blind, randomised controlled trial-THRIVE trial. | BMJ Open | Machin, M., S. Whittley, J. Norrie, L. Burgess, B. J. Hunt, L. Bolton, J. Shalhoub, T. Everington, M. Gohel, M. S. Whiteley, S. Rogers, S. Onida, B. Turner, S. Nandhra, R. Lawton, A. Stephens-Boal, C. Singer, J. Dunbar, D. Carradice and A. H. Davies | 10.1136/bmjopen-2023-083488 |
| 2024 | Ten-year outcomes of a randomized clinical trial of endothermal ablation versus conventional surgery for great saphenous varicose veins. | Br J Surg | Mohamed, A. H., A. Howitt, S. Rae, P. L. Cai, L. Hitchman, T. Wallace, S. Nandhra, S. Pymer, A. Knighton, G. Smith, I. C. Chetter and D. Carradice | 10.1093/bjs/znae195 |
| 2024 | Editor's Choice - Short Term Cost Effectiveness of Radiofrequency Ablation and High Ligation and Stripping for Great Saphenous Vein Incompetence. | Eur J Vasc Endovasc Surg | Nelzén, O., J. Skoog, L. Bernfort and H. Zachrisson | 10.1016/j.ejvs.2024.01.085 |
| 2024 | Endovenous laser ablation (EVLA) for vein insufficiency: two-year results of a multicenter experience with 1940-nm laser diode and a novel optical fiber. | Lasers Med Sci | Palombi, L., M. Morelli, D. Bruzzese, F. Martinelli, G. Quarto and P. G. Bianchi | 10.1007/s10103-024-04000-7 |
| 2024 | Efficacy and safety of crisaborole ointment, 2%, in participants aged ≥45 years with stasis dermatitis: Results from a fully decentralized, randomized, proof-of-concept phase 2a study. | J Am Acad Dermatol | Silverberg, J. I., R. S. Kirsner, D. J. Margolis, M. Tharp, D. E. Myers, K. Annis, D. Graham, C. Zang, B. L. Vlahos and P. Sanders | 10.1016/j.jaad.2023.12.048 |
| 2024 | Impact on healing of double-layered compression stocking in the treatment of severe venous leg ulcers: A prospective, multicenter, controlled trial. | J Med Vasc | Stansal, A., A. Marchand, I. Lazareth, U. Michon-Pasturel, A. Yannoutsos, C. Ostrowka, C. Berthin, T. Klejtman, N. Sigg, C. Jaillette, F. Perrinet, R. Attal, S. Sebbane, M. Pecourt, P. Ghaffari, C. Caucanas, E. Sacco, H. Beaussier and P. Priollet | 10.1016/j.jdmv.2024.07.001 |
| 2024 | Randomized controlled study on the application effect of a new type of intravenous radiofrequency closed therapy system made in China and an imported system. | Zhonghua Wai Ke Za Zhi | Tang, M. J., L. Y. Zhou, X. J. Jia, J. J. Wu, Y. B. Lou, M. J. Jin and Y. F. Zhu | 10.3760/cma.j.cn112139-20231012-00167 |
| 2024 | Varicose vein treatment reduces edema not only in the treated legs but also in the entire body. | Phlebology | Tomita, S., T. Mizukami, Y. Koyama, M. Inagaki, S. Ozoe and Y. Okawa | 10.1177/02683555241251647 |
| 2024 | Comparison of 1-day versus 3-day intravenous terlipressin in cirrhosis patients with variceal bleeding: A pilot randomised controlled trial. | Aliment Pharmacol Ther | Vaishnav, M., S. Biswas, A. Shenoy, P. Pathak, A. Anand, S. Swaroop, A. Aggrawal, U. Arora, A. Elhence, S. Jagannath, D. Gunjan, S. Kedia, A. K. Mishra, S. Gamanagatti, B. Nayak, P. Garg and Shalimar | 10.1111/apt.17868 |
| 2024 | Efficacy of Diosmin in Reducing Lower-Extremity Swelling and Pain After Total Knee Arthroplasty: A Randomized, Controlled Multicenter Trial. | J Bone Joint Surg Am | Wang, Q., Q. Jin, L. Cai, C. Zhao, P. Feng, J. Jia, W. Xu, Q. Qian, Z. Ding, J. Xu, C. Gu, S. Zhang, H. Shi, H. Ma, Y. Deng, T. Zhang, Y. Song, Q. Wang, Y. Zhang, X. Zhou, L. Pei, Y. Yang, J. Liang, T. Jiang, H. Li, H. Liu, L. Wu and P. Kang | 10.2106/jbjs.23.00854 |
| 2024 | Effect of Yoga in Industrial Workers with Chronic Venous Insufficiency: A Randomized Controlled Trial. | J Integr Complement Med | Yamuna, U., B. Pravalika, K. Madle, V. Majumdar and A. A. Saoji | 10.1089/jicm.2023.0691 |
| 2024 | A randomized controlled trial of standard vs customized graduated elastic compression stockings in patients with chronic venous disease. | J Vasc Surg Venous Lymphat Disord | Yang, W. T., Y. Xiong, S. X. Wang, H. L. Ren, C. Gong, Z. Y. Jin, J. H. Wen, W. D. Zhang, X. M. Tao and C. M. Li | 10.1016/j.jvsv.2023.08.017 |
| 2024 | Safety and efficacy analysis of a varicose vein sealant kit in the treatment of great saphenous vein dysfunction. | Zhonghua Yi Xue Za Zhi | Zou, S. L., K. K. Zhi, Y. Hong, L. R. Guo, Y. X. Qi, L. Zhang, J. L. Peng, B. Ye, G. F. Zheng, B. Hao, H. M. Xu, B. Chen, Y. F. Pan, Y. F. Zhu, J. J. Wu, X. J. Huang, S. C. Wen, X. Wang and L. F. Qu | 10.3760/cma.j.cn112137-20231208-01323 |
| 2023 | A comparison of patient-reported outcome measures following technical success and technical failure in the treatment of great saphenous vein incompetence using ClariVein: A subanalysis of a multicenter randomized controlled trial comparing 2% and 3% polidocanol. | Phlebology | Alozai, T., Y. L. Lam, M. A. Schreve, A. A. de Smet, A. C. Vahl, L. C. Terlouw-Punt, Ç. Ünlü and C. H. Wittens | 10.1177/02683555231189414 |
| 2023 | A randomized clinical trial to assess the impact of laser power with constant linear endovenous energy density on outcomes of endovenous laser ablation (SLEDGE trial). | J Vasc Surg Venous Lymphat Disord | Borsuk, D. A., A. A. Fokin, K. V. Lobastov, R. A. Tauraginskii, K. O. Zhdanov, A. V. Zolotov, I. S. Arkhipov and M. I. Galchenko | 10.1016/j.jvsv.2023.03.020 |
| 2023 | Ovarian vein surgical ablation versus endovascular technique for treatment of pelvic vein incompetence. | J Vasc Surg Venous Lymphat Disord | Emad El Din, M., M. Soliman, Y. El Kiran, S. Regal, H. Youssef, H. Elwakeel and R. Soliman | 10.1016/j.jvsv.2022.10.018 |
| 2023 | Comparison of endovenous laser ablation and cyanoacrylate embolization in the non-invasive treatment of superficial venous insufficiency in terms of patient satisfaction. | Phlebology | Er, Z. C., F. Ikbali Afsar, K. Atılgan and B. E. Onuk | 10.1177/02683555231182020 |
| 2023 | Nd:Yag laser combined with injection sclerotherapy in the treatment of reticular veins and telangiectasias (CLaCS method): A triple-blind randomized clinical trial comparing two sclerosing agents associated with same laser patterns. | Phlebology | Fonseca, M. M., F. J. Mocelin, M. H. Grill, S. Gianesini, K. Miyake, R. Argenta and A. H. Pereira | 10.1177/02683555231153533 |
| 2023 | FOVELASS: A Randomised Trial of Endovenous Laser Ablation Versus Polidocanol Foam for Small Saphenous Vein Incompetence. | Eur J Vasc Endovasc Surg | Hamel-Desnos, C., I. Nyamekye, B. Chauzat, S. Gracia, M. Josnin and F. Abbadie | 10.1016/j.ejvs.2022.11.021 |
| 2023 | Femoral nerve blockade during endovenous laser ablation of great saphenous vein decreases pain but does not affect the use of opioids during the procedure. | J Vasc Surg Venous Lymphat Disord | Hurmerinta-Kurkijärvi, O., E. M. Weselius, K. Halmesmäki, P. Vikatmaa, L. Vikatmaa and M. Venermo | 10.1016/j.jvsv.2023.04.007 |
| 2023 | A new compression stocking with well-defined pressure-a randomized controlled pilot study. | Phlebology | Källman, U., M. Fallenius and C. Bååth | 10.1177/02683555231200974 |
| 2023 | MINI-INVASIVE TREATMENT METHODS OF SPIDER VEINS: SCLEROTHERAPY AND RADIOFREQUENCY THERMOCOAGULATION. | Wiad Lek | Korolova, K., Z. Korolova, V. Teplyi and R. Sydorenko | 10.36740/WLek202309113 |
| 2023 | Ultrasound-guided femoral block in patients undergoing radiofrequency ablation of incompetent saphenous veins: A randomized controlled trial. | Asian J Surg | Lomarat, N., J. Akaraprasertkul, N. Wongchompoo, B. Boonsawek and N. Sermsathanasawadi | 10.1016/j.asjsur.2022.03.005 |
| 2023 | Comparison of carvedilol and propranolol for primary prophylaxis of esophageal variceal bleed in cirrhotic patients. | Pak J Pharm Sci | Muhammad Farooq, H., F. Raja Omer, I. Muhammad Adnan, I. Aneeza, Maria, K. Khalid Mahmud and G. Nooman | |
| 2023 | Six Year Extension Study of Patients From a Randomised Clinical Trial Comparing Venefit, Radiofrequency Induced Thermal Therapy, and Endovenous Radiofrequency Ablation for Treatment of Incompetent Great Saphenous Veins. | Eur J Vasc Endovasc Surg | Nyamekye, I. K., B. J. Pullen, N. Kelly and W. Hayes | 10.1016/j.ejvs.2023.03.021 |
| 2023 | A randomized clinical trial of isolated ambulatory phlebectomy versus saphenous thermal ablation with concomitant phlebectomy (SAPTAP Trial). | Br J Surg | Scheerders, E. R. Y., S. K. van der Velden, L. M. A. Goossens, S. A. S. Hamann, M. G. R. de Maeseneer, W. S. J. Malskat, L. de Mik, T. E. C. Nijsten and R. R. van den Bos | 10.1093/bjs/znac388 |
| 2023 | Randomized prospective comparative study of platelet-rich plasma versus conventional compression in treatment of post-phlebitic venous ulcer. | Vascular | Shehab, A. W., A. Eleshra, E. Fouda, H. Elwakeel and M. Farag | 10.1177/17085381221104629 |
| 2023 | Prevention of saphenous nerve injury after below-knee laser ablation of incompetent great saphenous veins: A trial of two-step ablation and an early result. | Phlebology | Utoh, J. and Y. Tsukamoto | 10.1177/02683555231183780 |
| 2023 | Endoscopic ultrasonography-guided injection of cyanoacrylate in the treatment of gastroesophageal varices type 1: a single-center randomized study. | Surg Endosc | Wang, Z., Z. Zeng, L. Chen, C. Shi, J. Jin, F. Zhang, Q. Zhang, X. Mei and D. Kong | 10.1007/s00464-023-10342-0 |
| 2023 | Comparison of day surgery between varicose veins with and without superficial venous thrombosis below knee: a propensity score-matched analysis. | BMC Cardiovasc Disord | Xu, J., X. Xu, J. Tian, M. Huang, Z. Xia, X. Luo, J. Zheng and K. Huang | 10.1186/s12872-023-03398-2 |
| 2023 | Prospective randomized trial of antithrombotic strategies following great saphenous vein ablation using injectable polidocanol endovenous microfoam (Varithena). | J Vasc Surg Venous Lymphat Disord | Yang, J., S. Chung and S. Srivatsa | 10.1016/j.jvsv.2022.12.008 |
| 2022 | Radiofrequency ablation for markedly incompetent perforators versus compression therapy in the management of post-phelebtic venous ulcers: A randomized controlled trial. | Vascular | Abdelgawad, M. S., A. M. El-Shafei, H. A. Sharaf El-Din, E. M. Saad, T. A. Khafagy, A. Sameer, N. A. Elsaadany and M. A. Abdelmaksoud | 10.1177/17085381211010022 |
| 2022 | Effects of inspiratory muscle training versus calf muscle training on quality of life, pain, venous function and activity in patients with chronic venous insufficiency. | J Vasc Surg Venous Lymphat Disord | Aydin, G., I. Yeldan, A. Akgul and G. Ipek | 10.1016/j.jvsv.2022.04.012 |
| 2022 | The effectiveness of different treatment methods in isolated telangiectasia and reticular vein treatment: A single-center prospective randomized study. | Phlebology | Aydın, U., M. Engin, T. Türk and Y. Ata | 10.1177/02683555211030739 |
| 2022 | Pain Outcomes Following Mechanochemical Ablation vs Cyanoacrylate Adhesive for the Treatment of Primary Truncal Saphenous Vein Incompetence: The MOCCA Randomized Clinical Trial. | JAMA Surg | Belramman, A., R. Bootun, T. Y. Tang, T. R. A. Lane and A. H. Davies | 10.1001/jamasurg.2022.0298 |
| 2022 | Radio wave electrotherapy with a radiofrequency of 448 khz for the treatment of patients with organic erectile dysfunction: a prospective, randomized, blind, Sham-controlled, parallel-group study. | Urologiia | Chuvalov, L. L., D. O. Korolev, K. R. Azilgareeva, M. S. Taratkin, Y. V. Olefir, D. N. Fiev, I. S. Lumpov, Y. P. Gorobets, D. V. Enikeev, L. M. Rapoport and M. E. Enikeev | |
| 2022 | Ten-year follow-up of a randomized controlled trial comparing saphenofemoral ligation and stripping of the great saphenous vein with endovenous laser ablation (980 nm) using local tumescent anesthesia. | J Vasc Surg Venous Lymphat Disord | Eggen, C. A. M., T. Alozai, P. Pronk, M. C. Mooij, M. T. W. Gaastra, Ç. Ünlü, M. A. Schreve and C. J. van Vlijmen | 10.1016/j.jvsv.2021.08.008 |
| 2022 | Safety of High-Intensity, Low-Volume Interval Training or Continuous Aerobic Training in Adults With Metabolic Syndrome. | J Patient Saf | Gallo-Villegas, J., D. Restrepo, L. Pérez, L. A. Castro-Valencia, R. Narvaez-Sanchez, J. Osorio, D. C. Aguirre-Acevedo and J. C. Calderón | 10.1097/pts.0000000000000922 |
| 2022 | A multicenter randomized controlled trial of cyanoacrylate closure and surgical stripping for incompetent great saphenous veins. | J Vasc Surg Venous Lymphat Disord | Joh, J. H., T. Lee, S. J. Byun, S. Cho, H. S. Park, W. S. Yun, S. S. Yang, H. Kim, W. S. Kim and I. M. Jung | 10.1016/j.jvsv.2021.08.012 |
| 2022 | A prospective multicenter randomized clinical trial comparing endovenous laser ablation, using a 1470 nm diode laser in combination with a Tulip-TipTM fiber versus radiofrequency (Closure FAST™ VNUS®), in the treatment of primary varicose veins. | Int Angiol | Kempeneers, A. C., B. Bechter-Hugl, S. Thomis, D. van den Bussche, M. E. Vuylsteke and M. M. Vuylsteke | 10.23736/s0392-9590.22.04747-2 |
| 2022 | Axial ablation versus terminal interruption of the reflux source (AAVTIRS): a randomised controlled trial. | Trials | Keohane, C. R., D. Westby, M. Twyford, T. Ahern, W. Tawfick and S. R. Walsh | 10.1186/s13063-022-06440-4 |
| 2022 | Non-drug methods of treatment of post-thrombophlebitic syndrome. | Vopr Kurortol Fizioter Lech Fiz Kult | Kulchitskaya, D. B., A. D. Fesyun, T. V. Apkhanova, T. V. Konchugova, M. Y. Yakovlev, N. V. Gushchina and O. M. Musaeva | 10.17116/kurort20229905122 |
| 2022 | A multicenter, randomized, dose-finding study of mechanochemical ablation using ClariVein and liquid polidocanol for great saphenous vein incompetence. | J Vasc Surg Venous Lymphat Disord | Lam, Y. L., T. Alozai, M. A. Schreve, A. de Smet, A. C. Vahl, I. Nagtzaam, J. A. Lawson, F. H. M. Nieman and C. H. A. Wittens | 10.1016/j.jvsv.2021.10.016 |
| 2022 | Concurrent large spontaneous portosystemic shunt embolization for the prevention of overt hepatic encephalopathy after TIPS: A randomized controlled trial. | Hepatology | Lv, Y., H. Chen, B. Luo, W. Bai, K. Li, Z. Wang, D. Xia, W. Guo, Q. Wang, X. Li, J. Yuan, H. Cai, J. Xia, Z. Yin, D. Fan and G. Han | 10.1002/hep.32453 |
| 2022 | The Effect of Active Stretching Training in Patients with Chronic Venous Insufficiency Monitored by Raster-Stereography. | Sensors (Basel) | Menegatti, E., S. Mandini, A. Pagani, B. Mandini, V. Zerbini, T. Piva, A. Raisi, M. Fabbri, M. Fogli, G. Mazzoni, P. Zamboni and S. Gianesini | 10.3390/s22218509 |
| 2022 | Effects of acupuncture on pain and levels of IL-17 and IL-23 in the treatment of non-thermal endovenous ablation: A randomized clinical trial. | Vascular | Orak, Y., E. Eroğlu, F. A. Baylan, S. Yıldız, F. Boran Ö, A. Doganer and A. Altun | 10.1177/17085381211013980 |
| 2022 | Predictors of the Level of Shared Decision Making in Vascular Surgery: A Cross Sectional Study. | Eur J Vasc Endovasc Surg | Peters, L. J., F. E. Stubenrouch, J. B. Thijs, P. L. Klemm, R. Balm and D. T. Ubbink | 10.1016/j.ejvs.2022.05.002 |
| 2022 | Recovery and patient satisfaction following radiofrequency ablation and concomitant foam sclerotherapy of varicose veins with and without compression: A randomized controlled non-inferiority trial. | Phlebology | Pihlaja, T., M. Mella, P. Ohtonen, P. Romsi and M. Pokela | 10.1177/02683555221077742 |
| 2022 | Factors associated with delayed venous ulcer healing after endovenous intervention for superficial venous insufficiency. | J Vasc Surg Venous Lymphat Disord | Pihlaja, T., L. M. Vanttila, P. Ohtonen and M. Pokela | 10.1016/j.jvsv.2022.07.008 |
| 2022 | An Open-Label, Prospective, Pilot Study of Hypertonic Saline for Hidradenitis Suppurativa. | Dermatol Surg | Porter, M. L., P. Salian, M. Rosales Santillan, C. Greif and A. B. Kimball | 10.1097/dss.0000000000003510 |
| 2022 | Ultrasound-assisted varicose vein surgery and endovenous laser ablation using 1470-nm laser for treatment of great saphenous vein incompetence has similar outcomes at 1 year in a single-center prospective randomized study. | J Vasc Surg Venous Lymphat Disord | Rajendran, S., H. R. Nair, K. M. Irshad, T. M. Unais and N. J. Thaikattil | 10.1016/j.jvsv.2021.08.013 |
| 2022 | Lower prevalence of stump reflux after endovenous laser flush ablation of the great saphenous vein. | Vasa | Rits, J., U. Maurins, E. Rabe, A. Kadiss, S. Prave, R. Vigants, I. Brunenieks and F. Pannier | 10.1024/0301-1526/a001007 |
| 2022 | Safety and efficacy of endovenous laser ablation (EVLA) using 1940 nm and radial emitting fiber: 3-year results of a prospective, non-randomized study and comparison with 1470 nm. | Lasers Surg Med | Setia, A., C. G. Schmedt, A. Beisswenger, S. Dikic, S. Demhasaj, O. Setia, T. Schmitz-Rixen and R. Sroka | 10.1002/lsm.23500 |
| 2022 | Propranolol vs. band ligation for primary prophylaxis of variceal hemorrhage in cirrhotic patients with ascites: a randomized controlled trial. | Hepatol Int | Singh, V., P. Kumar, N. Verma, R. Vijayvergiya, A. Singh and A. Bhalla | 10.1007/s12072-022-10361-4 |
| 2022 | Improving Shared Decision Making in Vascular Surgery: A Stepped Wedge Cluster Randomised Trial. | Eur J Vasc Endovasc Surg | Stubenrouch, F. E., L. J. Peters, S. M. L. de Mik, P. L. Klemm, A. G. Peppelenbosch, S. Schreurs, D. M. Scharn, D. A. Legemate, R. Balm and D. T. Ubbink | 10.1016/j.ejvs.2022.04.016 |
| 2022 | Predictors of Clinical Outcomes of Pharmacomechanical Catheter-Directed Thrombolysis for Acute Iliofemoral Deep Vein Thrombosis: Analysis of a Multicenter Randomized Trial. | J Vasc Interv Radiol | Thukral, S., A. Salter, S. Lancia, S. R. Kahn and S. Vedantham | 10.1016/j.jvir.2022.05.030 |
| 2022 | A randomized controlled trial to evaluate the safety and efficacy of transluminal injection of foam sclerotherapy compared with ultrasound-guided foam sclerotherapy during endovenous catheter ablation in patients with varicose veins. | J Vasc Surg Venous Lymphat Disord | Watanabe, S., A. Okamura, M. Iwamoto, H. Nagai, A. Sumiyoshi, K. Tanaka, S. Suzuki, H. Tanaka, K. Iwakura and K. Fujii | 10.1016/j.jvsv.2021.06.017 |
| 2022 | Predictive value of hepatic venous pressure gradient and efficacy and significance of early PTVE for gastrointestinal bleeding after TACE for liver cancer. | J Cancer Res Ther | Wei, J., Y. Hu, J. Yu, C. Yin, G. Chen and L. Jin | 10.4103/jcrt.jcrt_331_22 |
| 2022 | Reducing tumescent anesthetic injection pain by topical anesthesia pretreatment among patients undergoing endovenous radiofrequency ablation of varicose veins: A double-blind randomized controlled trial. | Phlebology | Zhang, Y. S., P. J. Chen, H. L. Wan, J. H. Chen, T. Mei, W. L. Wang and Y. M. Lu | 10.1177/02683555221092193 |
| 2021 | Correlation between great saphenous length of treatment zone and diameter with improvement in symptoms after ablation. | J Vasc Surg Venous Lymphat Disord | Attaran, R. R., A. Bhalla, C. I. Mena-Hurtado and C. I. Ochoa Chaar | 10.1016/j.jvsv.2021.02.013 |
| 2021 | Clinical outcome of short-term compression after sclerotherapy for telangiectatic varicose veins. | J Vasc Surg Venous Lymphat Disord | Bayer, A., N. Kuznik, E. A. Langan, A. Recke, A. L. Recke, G. Faerber, M. Kaschwich, M. Kleemann and B. Kahle | 10.1016/j.jvsv.2020.05.015 |
| 2021 | Randomized Controlled Trial of Compression After Endovenous Thermal Ablation of Varicose Veins (COMETA Trial). | Ann Surg | Bootun, R., A. Belramman, L. Bolton-Saghdaoui, T. R. A. Lane, C. Riga and A. H. Davies | 10.1097/sla.0000000000003626 |
| 2021 | Compression Stocking With 100% Donning and Doffing Success: An Open Label Randomised Controlled Trial. | Eur J Vasc Endovasc Surg | Buset, C. S., J. Fleischer, R. Kluge, N. T. Graf, G. Mosti, H. Partsch, C. Seeli, F. Anzengruber, M. Kockaert, M. Hübner and J. Hafner | 10.1016/j.ejvs.2020.09.027 |
| 2021 | Adjuvant radiofrequency thermocoagulation improves the outcome of liquid sclerotherapy in the treatment of spider veins of the leg: A pilot study. | Phlebology | Diken, A., U. Alemdaroğlu, S. Özyalçın, İ. Hafez, H. A. Tünel, A. Yalçınkaya and A. N. Ecevit | 10.1177/02683555211006534 |
| 2021 | Data Recorded in Real Life Support the Safety of Nattokinase in Patients with Vascular Diseases. | Nutrients | Gallelli, G., G. Di Mizio, C. Palleria, A. Siniscalchi, P. Rubino, L. Muraca, E. Cione, M. Salerno, G. De Sarro and L. Gallelli | 10.3390/nu13062031 |
| 2021 | A randomized controlled noninferiority trial comparing radiofrequency with stripping and conservative hemodynamic cure for venous insufficiency technique for insufficiency of the great saphenous vein. | J Vasc Surg Venous Lymphat Disord | González Cañas, E., S. Florit López, R. V. Vilagut, K. A. Guevara-Noriega, M. Santos Espí, J. Rios, S. N. Soto and A. Giménez Gaibar | 10.1016/j.jvsv.2020.04.019 |
| 2021 | Reducing hyperpigmentation after sclerotherapy: A randomized clinical trial. | J Vasc Surg Venous Lymphat Disord | Gonzalez Ochoa, A. J., J. Carrillo, D. Manríquez, F. Manrique and A. N. Vazquez | 10.1016/j.jvsv.2020.06.019 |
| 2021 | The efficiency of exercise training in patients with venous insufficiency: A double blinded, randomized controlled trial. | Phlebology | Gürdal Karakelle, S., Y. Ipek, O. Tulin and U. Alpagut İ | 10.1177/0268355520985759 |
| 2021 | A NEW MIXED SURGICAL TREATMENT FOR GRADES III AND IV HEMORRHOIDS: MODIFIED SELECTIVE HEMORRHOIDECTOMY COMBINED WITH COMPLETE ANAL EPITHELIAL RETENTION. | Arq Bras Cir Dig | Huang, H., Y. Gu, L. Ji, Y. Li, S. Xu, T. Guo and M. Xu | 10.1590/0102-672020210002e1594 |
| 2021 | Intermittent pneumatic compression after varicose vein surgery. | J Vasc Surg Venous Lymphat Disord | Kappa-Markovi, K., H. Jalaie, H. Özhan-Hasan, M. Deges and K. Rass | 10.1016/j.jvsv.2021.02.011 |
| 2021 | Randomized clinical trial of radiofrequency-induced thermotherapy combined with transilluminated powered phlebectomy versus high ligation and stripping for the treatment of lower limb varicose veins. | J Vasc Surg Venous Lymphat Disord | Liao, C. J., S. H. Song, T. Li, Y. Zhang and W. D. Zhang | 10.1016/j.jvsv.2020.04.028 |
| 2021 | Registry to investigate the efficacy and safety of the VenaBlock(©) VeIn SEaling system for VaRicose veins in SingApore - Six months results of the RIVIERA trial. | Phlebology | Linn, Y. L., C. Yap, S. Soon, S. L. Chan, V. Khoo, T. T. Chong and T. Y. Tang | 10.1177/02683555211025181 |
| 2021 | Influence of Medical Compression Stockings on Skin Hydration in Mainly Health Care Givers with Occupational Leg Symptoms and Edema. | Skin Pharmacol Physiol | Mayer-Yousif, M., W. Konschake, H. Haase, M. Jünger and H. Riebe | 10.1159/000512642 |
| 2021 | Randomized controlled trial on Dryland And Thermal Aquatic standardized exercise protocol for chronic venous disease (DATA study). | J Vasc Surg Venous Lymphat Disord | Menegatti, E., S. Masiero, P. Zamboni, G. Avruscio, M. Tessari, A. Pagani and S. Gianesini | 10.1016/j.jvsv.2020.12.078 |
| 2021 | A Randomized Controlled Trial of Endovenous Laser Ablation Versus Mechanochemical Ablation With ClariVein in the Management of Superficial Venous Incompetence (LAMA Trial). | Ann Surg | Mohamed, A. H., C. Leung, T. Wallace, G. Smith, D. Carradice and I. Chetter | 10.1097/sla.0000000000003749 |
| 2021 | Endovenous laser ablation of incompetent great saphenous veins with 1470-nm laser using bare tip and radial fibers results in similar short-term outcomes. | J Vasc Surg Venous Lymphat Disord | Rajendran, S. and H. R. Nair | 10.1016/j.jvsv.2020.12.069 |
| 2021 | A randomised controlled trial of neuromuscular stimulation in non-operative venous disease improves clinical and symptomatic status. | Phlebology | Ravikumar, R., T. R. Lane, A. Babber, S. Onida and A. H. Davies | 10.1177/0268355520968640 |
| 2021 | The randomized, controlled ATLANTIS trial of aquatic therapy for chronic venous insufficiency. | J Vasc Surg Venous Lymphat Disord | Sharifi, M., R. C. Bay, K. Karandish, F. Emrani, R. Snyder and S. D'Silva | 10.1016/j.jvsv.2020.10.016 |
| 2021 | Bromelain-based enzymatic debridement of chronic wounds: Results of a multicentre randomized controlled trial. | Wound Repair Regen | Shoham, Y., E. Shapira, J. Haik, M. Harats, D. Egozi, D. Robinson, L. Kogan, R. Elkhatib, G. Telek and A. Shalom | 10.1111/wrr.12958 |
| 2021 | Three-year results of a randomized controlled trial comparing mechanochemical and thermal ablation in the treatment of insufficient great saphenous veins. | J Vasc Surg Venous Lymphat Disord | Vähäaho, S., K. Halmesmäki, O. Mahmoud, A. Albäck, K. Noronen and M. Venermo | 10.1016/j.jvsv.2020.08.007 |
| 2021 | Safety and feasibility report on nonimplantable endovenous valve formation for the treatment of deep vein reflux. | J Vasc Surg Venous Lymphat Disord | Vasudevan, T., D. A. Robinson, A. A. Hill, K. Ouriel, A. Holden, J. Gagnon, L. Machan, I. Nammuni, S. D. Thomas and R. L. Varcoe | 10.1016/j.jvsv.2020.12.073 |
| 2021 | Clinical Outcomes of a Pharmacomechanical Catheter-Directed Venous Thrombolysis Strategy that Included Rheolytic Thrombectomy in a Multicenter Randomized Trial. | J Vasc Interv Radiol | Vedantham, S., A. Salter, S. Lancia, L. Lewis, S. Thukral and S. R. Kahn | 10.1016/j.jvir.2021.06.001 |
| 2021 | Once daily 300 mg aspirin with compression versus compression alone in patients with chronic venous leg ulcers (ASPiVLU): A randomised, double-blinded, multicentre, placebo-controlled, clinical trial. | J Tissue Viability | Weller, C. D., C. Martin, A. Bouguettaya, M. Underwood, A. L. Barker, T. Haines, D. Pouniotis and R. Wolfe | 10.1016/j.jtv.2021.07.005 |
| 2021 | A randomized trial of class II compression sleeves for full legs versus stockings after thermal ablation with phlebectomy. | J Vasc Surg Venous Lymphat Disord | Zolotukhin, I., M. Demekhova, E. Ilyukhin, I. Sonkin, E. Zakharova, O. Efremova, E. Kiseleva and E. Gavrilov | 10.1016/j.jvsv.2020.12.067 |
| 2020 | The effect of natural matrix biopolymer membrane on hard-to-heal venous leg ulcers: a pilot randomised clinical trial. | J Wound Care | Atias, Z., J. M. Pederson, H. K. Mishra and S. Greenberger | 10.12968/jowc.2020.29.5.295 |
| 2020 | Ablation of the great saphenous vein with F-care versus Closurefast endovenous radiofrequency therapy: Double-blinded prospective study. | Phlebology | Bitargil, M. and H. E. Kılıç | 10.1177/0268355520913389 |
| 2020 | Elastic compression after ultrasound-guided foam sclerotherapy in overweight patients does not improve primary venous hemodynamics outcomes. | J Vasc Surg Venous Lymphat Disord | Campos Gomes, C. V., M. A. Prado Nunes, T. P. Navarro and A. Dardik | 10.1016/j.jvsv.2019.07.008 |
| 2020 | Comparison of high ligation of great saphenous vein using pneumatic tourniquets and conventional method for great saphenous vein varicosis. | Medicine (Baltimore) | Chen, P., H. Chen and M. Yang | 10.1097/md.0000000000021975 |
| 2020 | Sulodexide in the Treatment of Chronic Venous Insufficiency: Results of the All-Russian Multicenter ACVEDUCT Program. | Adv Ther | Chupin, A. V., S. E. Katorkin, Katelnitsky, II, O. V. Katelnitskaya, Prostov, II, A. S. Petrikov, A. P. Koshevoi and L. F. Lyudkova | 10.1007/s12325-020-01270-9 |
| 2020 | Improving shared decision-making in vascular surgery by implementing decision support tools: study protocol for the stepped-wedge cluster-randomised OVIDIUS trial. | BMC Med Inform Decis Mak | de Mik, S. M. L., F. E. Stubenrouch, D. A. Legemate, R. Balm and D. T. Ubbink | 10.1186/s12911-020-01186-y |
| 2020 | Dancing: More than a therapy for patients with venous insufficiency. | Vascular | Dogru-Huzmeli, E., I. Fansa, N. Cetisli-Korkmaz, G. Oznur-Karabicak, C. Lale, O. Gokcek and Y. Cam | 10.1177/1708538119893534 |
| 2020 | Catheter-directed foam sclerotherapy with tumescence of the great saphenous vein versus ultrasound-guided foam sclerotherapy: A randomized controlled trial. | Phlebology | Dos Santos, J. B., W. C. Júnior, R. M. Porta, J. Puggina, D. F. da Silva, P. Puech-Leão, N. de Luccia and E. S. da Silva | 10.1177/0268355519850651 |
| 2020 | Long-term risk of postthrombotic syndrome after symptomatic distal deep vein thrombosis: The CACTUS-PTS study. | J Thromb Haemost | Galanaud, J. P., M. Righini, L. Le Collen, A. Douillard, H. Robert-Ebadi, D. Pontal, D. Morrison, M. T. Barrellier, A. Diard, H. Guénnéguez, D. Brisot, P. Faïsse, S. Accassat, M. Martin, A. Delluc, S. Solymoss, J. Kassis, M. Carrier, I. Quéré and S. R. Kahn | 10.1111/jth.14728 |
| 2020 | Ultrasound-Accelerated Thrombolysis and Venoplasty for the Treatment of the Postthrombotic Syndrome: Results of the ACCESS PTS Study. | J Am Heart Assoc | Garcia, M. J., K. M. Sterling, S. R. Kahn, A. J. Comerota, M. R. Jaff, K. Ouriel and I. Weinberg | 10.1161/jaha.119.013398 |
| 2020 | A prospective safety and effectiveness study using endovenous laser ablation with a 400-μm optical fiber for the treatment of pathologic perforator veins in patients with advanced venous disease (SeCure trial). | J Vasc Surg Venous Lymphat Disord | Gibson, K., S. Elias, M. Adelman, E. S. Hager, D. J. Dexter, S. Vayuvegula, P. Chopra and L. S. Kabnick | 10.1016/j.jvsv.2020.01.014 |
| 2020 | Effects of the use of bioceramic wraps in patients with lower limb venous ulcers: A randomized double-blind placebo-controlled trial. | J Integr Med | Herr, G. E. G., F. G. da Silva, F. J. Cidral-Filho, F. Petronilho, L. G. Danielski, M. P. de Souza Goldim, A. S. I. Salgado, F. Bobinski, D. F. Martins and E. R. Winkelmann | 10.1016/j.joim.2019.11.006 |
| 2020 | Efficacy and mechanism of fire needling bloodletting for lower extremity varicose veins. | Zhongguo Zhen Jiu | Jiang, H., L. L. Qiu, Y. Y. Li, L. P. Gu and Q. G. Liu | 10.13703/j.0255-2930.20190815-k0004 |
| 2020 | Open-label, randomised, multicentre crossover trial assessing two-layer compression bandaging for chronic venous insufficiency: results of the APRICOT trial. | Br J Community Nurs | Jonker, L., J. Todhunter, L. Robinson and S. Fisher | 10.12968/bjcn.2020.25.Sup6.S6 |
| 2020 | Compression stocking prevents increased venous retrograde flow time in the lower limbs of pregnant women. | Phlebology | Junior, O. A. S., H. A. Rollo, O. Saliba and M. L. Sobreira | 10.1177/0268355520939371 |
| 2020 | Quality of life after pharmacomechanical catheter-directed thrombolysis for proximal deep venous thrombosis. | J Vasc Surg Venous Lymphat Disord | Kahn, S. R., J. A. Julian, C. Kearon, C. S. Gu, D. J. Cohen, E. A. Magnuson, A. J. Comerota, S. Z. Goldhaber, M. R. Jaff, M. K. Razavi, A. L. Kindzelski, J. R. Schneider, P. Kim, R. Chaer, A. K. Sista, R. B. McLafferty, J. A. Kaufman, B. C. Wible, M. Blinder and S. Vedantham | 10.1016/j.jvsv.2019.03.023 |
| 2020 | Great saphenous vein sparing versus stripping in Trendelenburg operation for primary varicose veins: a prospective study. | Pol Przegl Chir | Kundal, A., N. Kumar, D. Rajput and U. Chauhan | 10.5604/01.3001.0014.6219 |
| 2020 | Economic benefit of a novel dual-mode ambulatory compression device for treatment of chronic venous leg ulcers in a randomized clinical trial. | J Vasc Surg Venous Lymphat Disord | Marston, W. A., R. S. Kirsner, A. Tallis, J. R. Hanft, J. Walters and A. Farber | 10.1016/j.jvsv.2020.03.004 |
| 2020 | Five-year extension study of patients from a randomized clinical trial (VeClose) comparing cyanoacrylate closure versus radiofrequency ablation for the treatment of incompetent great saphenous veins. | J Vasc Surg Venous Lymphat Disord | Morrison, N., K. Gibson, M. Vasquez, R. Weiss and A. Jones | 10.1016/j.jvsv.2019.12.080 |
| 2020 | Effects of Kinesio Taping and compression stockings on pain, edema, functional capacity and quality of life in patients with chronic venous disease: a randomized controlled trial. | Clin Rehabil | Naci, B., S. Ozyilmaz, N. Aygutalp, R. Demir, G. Baltaci and Z. Yigit | 10.1177/0269215520916851 |
| 2020 | A randomised controlled trial of perivenous tumescent anaesthesia in addition to general anaesthesia for surgical ligation and stripping of the great saphenous vein. | Phlebology | Nandhra, S., T. Wallace, J. El-Sheikha, D. Carradice and I. Chetter | 10.1177/0268355519885221 |
| 2020 | Percutaneous Venous Angioplasty in Patients with Multiple Sclerosis and Chronic Cerebrospinal Venous Insufficiency: A Randomized Wait List Control Study. | Ann Vasc Surg | Napoli, V., R. Berchiolli, M. C. Carboncini, F. Sartucci, M. Marconi, T. Bocci, O. Perrone, N. Mannoni, C. Congestrì, R. Benedetti, R. Morganti, D. Caramella, R. Cioni and M. Ferrari | 10.1016/j.avsg.2019.05.018 |
| 2020 | Impact on venous haemodynamics after treatment of great saphenous vein incompetence using plethysmography and duplex ultrasound. | Phlebology | Nelzén, P. O. E., J. Skoog, M. Öster and H. Zachrisson | 10.1177/0268355519898952 |
| 2020 | Editor's Choice - Role of Compression After Radiofrequency Ablation of Varicose Veins: A Randomised Controlled Trial(☆). | Eur J Vasc Endovasc Surg | Onwudike, M., K. Abbas, P. Thompson and D. M. McElvenny | 10.1016/j.ejvs.2020.03.014 |
| 2020 | Predictors of exercise capacity in chronic venous disease patients. | Phlebology | Ozberk, S., D. Karadibak and M. Polat | 10.1177/0268355519870895 |
| 2020 | Post-procedural Compression vs. No Compression After Radiofrequency Ablation and Concomitant Foam Sclerotherapy of Varicose Veins: A Randomised Controlled Non-inferiority Trial. | Eur J Vasc Endovasc Surg | Pihlaja, T., P. Romsi, P. Ohtonen, J. Jounila and M. Pokela | 10.1016/j.ejvs.2019.08.020 |
| 2020 | Results of purified micronized flavonoid fraction in the treatment of categorized type III chronic pelvic pain syndrome: a randomized controlled trial. | Aging Male | Sahin, A., M. A. Kutluhan, C. Yildirim, A. Urkmez, S. Akan and A. Verit | 10.1080/13685538.2019.1678581 |
| 2020 | Graduated compression stockings effects on chronic venous disease signs and symptoms during pregnancy. | Phlebology | Saliba Júnior, O. A., H. A. Rollo, O. Saliba and M. L. Sobreira | 10.1177/0268355519846740 |
| 2020 | Randomised controlled study to compare radiofrequency ablation with minimally invasive ultrasound-guided non-flush ligation and stripping of great saphenous vein in the treatment of varicose veins. | Ann R Coll Surg Engl | Sandhya, P. A., R. S. Mohil and R. Sricharan | 10.1308/rcsann.2020.0116 |
| 2020 | Laser ablation versus mechanochemical ablation in the treatment of primary varicose veins: A randomized clinical trial. | J Vasc Surg Venous Lymphat Disord | Tawfik, A. M., W. A. Sorour and M. E. El-Laboudy | 10.1016/j.jvsv.2019.10.025 |
| 2020 | Five-year outcomes of mechano-chemical ablation of primary great saphenous vein incompetence. | Phlebology | Thierens, N., S. Holewijn, W. H. Vissers, D. A. Werson, J. P. P. de Vries and M. M. Reijnen | 10.1177/0268355519861464 |
| 2020 | Effects of Venous Angioplasty on Cerebral Lesions in Multiple Sclerosis: Expanded Analysis of the Brave Dreams Double-Blind, Sham-Controlled Randomized Trial. | J Endovasc Ther | Zamboni, P., R. Galeotti, F. Salvi, A. Giaquinta, C. Setacci, S. Alborino, G. Guzzardi, S. J. Sclafani, E. Maietti and P. Veroux | 10.1177/1526602819890110 |
| 2019 | Gonarthrosis concurrent with chronic venous insufficiency: a new look at therapy. | Vopr Kurortol Fizioter Lech Fiz Kult | Ageeva, A. I., A. G. Kulikov, S. A. Volovets, M. Y. Gerasimenko and O. V. Yarustovskaya | 10.17116/kurort20199605129 |
| 2019 | Design and evaluation of the psychometric properties of a self-questionnaire on patient adherence to wearing elastic compression stockings. | Phlebology | Allaert, F. A., D. Rastel, A. Graissaguel, D. Sion and C. Hamel-Desnos | 10.1177/0268355518762824 |
| 2019 | Impact of copper compression stockings on venous insufficiency and lipodermatosclerosis: A randomised controlled trial. | Phlebology | Arendsen, L. P., S. Vig, R. Thakar and A. H. Sultan | 10.1177/0268355518795329 |
| 2019 | Drug-Coated Versus Plain Balloon Angioplasty In Arteriovenous Fistulas: A Randomized, Controlled Study With 1-Year Follow-Up (The Drecorest Ii-Study). | Scand J Surg | Björkman, P., E. M. Weselius, T. Kokkonen, V. Rauta, A. Albäck and M. Venermo | 10.1177/1457496918798206 |
| 2019 | Exercise and Chronic Wound Healing. | Wounds | Bolton, L. |  |
| 2019 | Five-Year Outcomes of a Randomized Trial of Treatments for Varicose Veins. | N Engl J Med | Brittenden, J., D. Cooper, M. Dimitrova, G. Scotland, S. C. Cotton, A. Elders, G. MacLennan, C. R. Ramsay, J. Norrie, J. M. Burr, B. Campbell, P. Bachoo, I. Chetter, M. Gough, J. Earnshaw, T. Lees, J. Scott, S. A. Baker, E. Tassie, J. Francis and M. K. Campbell | 10.1056/NEJMoa1805186 |
| 2019 | Ablation therapy with cyanoacrylate glue and laser for refluxing great saphenous veins - a prospective randomised study. | Vasa | Çalık, E. S., Ü. Arslan and B. Erkut | 10.1024/0301-1526/a000792 |
| 2019 | Compression with 23 mmHg or 35 mmHg stockings after saphenous catheter foam sclerotherapy and phlebectomy of varicose veins: A randomized controlled study. | Phlebology | Cavezzi, A., G. Mosti, R. Colucci, V. Quinzi, L. Bastiani and S. U. Urso | 10.1177/0268355518776127 |
| 2019 | Transilluminated powered phlebectomy in the treatment of large area venous leg ulcers: A case-control study with 3 years follow-up. | Microcirculation | Chen, S., Q. Zeng, Q. Fu, F. Li, M. Zhang and Y. Zhao | 10.1111/micc.12523 |
| 2019 | Effects of compression therapy and venous surgery on tissue oxygenation in chronic venous disease. | Phlebology | Chiang, N., O. Rodda, S. Oldham, J. Sleigh and T. Vasudevan | 10.1177/0268355518822582 |
| 2019 | Comparison of the effects of endovenous laser ablation at 1470 nm versus 1940 nm and different energy densities. | Phlebology | de Araujo, W. J. B., J. R. R. Timi, L. R. Kotze and C. R. Vieira da Costa | 10.1177/0268355518778488 |
| 2019 | Effect of Diosmin Administration in Patients with Chronic Venous Disorders on Selected Factors Affecting Angiogenesis. | Molecules | Feldo, M., M. Wójciak-Kosior, I. Sowa, J. Kocki, J. Bogucki, T. Zubilewicz, J. Kęsik and A. Bogucka-Kocka | 10.3390/molecules24183316 |
| 2019 | Need for adjunctive procedures following cyanoacrylate closure of incompetent great, small and accessory saphenous veins without the use of postprocedure compression: Three-month data from a postmarket evaluation of the VenaSeal System (the WAVES Study). | Phlebology | Gibson, K., R. Minjarez, K. Gunderson and B. Ferris | 10.1177/0268355518801641 |
| 2019 | Better wearing comfort of knee-length elastic compression stockings with an interface pressure of 18-21 mmHg compared to 23-32 mmHg in elderly people after a one day trial - Influence on foot deformities, rheumatism and arthritis. | Clin Hemorheol Microcirc | Goetz, J., E. Kaisermayer, H. Haase, M. Jünger and H. Riebe | 10.3233/ch-199207 |
| 2019 | Early versus deferred endovenous ablation of superficial venous reflux in patients with venous ulceration: the EVRA RCT. | Health Technol Assess | Gohel, M. S., F. Heatley, X. Liu, A. Bradbury, R. Bulbulia, N. Cullum, D. M. Epstein, I. Nyamekye, K. R. Poskitt, S. Renton, J. Warwick and A. H. Davies | 10.3310/hta23240 |
| 2019 | Randomized clinical trial of endovenous laser ablation versus direct and indirect radiofrequency ablation for the treatment of great saphenous varicose veins. | Br J Surg | Hamann, S. A. S., L. Timmer-de Mik, W. M. Fritschy, G. R. R. Kuiters, T. E. C. Nijsten and R. R. van den Bos | 10.1002/bjs.11187 |
| 2019 | Two-year results of a multicenter randomized controlled trial comparing Mechanochemical endovenous Ablation to RADiOfrequeNcy Ablation in the treatment of primary great saphenous vein incompetence (MARADONA trial). | J Vasc Surg Venous Lymphat Disord | Holewijn, S., R. van Eekeren, A. Vahl, J. de Vries and M. Reijnen | 10.1016/j.jvsv.2018.12.014 |
| 2019 | Compression therapy following ClariVein® ablation therapy: a randomised controlled trial of COMpression Therapy Following MechanO-Chemical Ablation (COMMOCA). | Trials | Joyce, D. P., S. R. Walsh, C. J. Q. Yap, T. T. Chong and T. Y. Tang | 10.1186/s13063-019-3787-4 |
| 2019 | The femoral vein diameter and its correlation with sex, age and body mass index - An anatomical parameter with clinical relevance. | Phlebology | Keiler, J., R. Seidel and A. Wree | 10.1177/0268355518772746 |
| 2019 | A randomised controlled trial comparing compression therapy after stripping for primary great saphenous vein incompetence. | Phlebology | Krasznai, A. G., T. A. Sigterman, J. P. Houtermans-Auckel, E. Eussen, M. Snoeijs, K. J. J. Sikkink, E. M. de Boer, C. H. Wittens and L. H. Bouwman | 10.1177/0268355519833255 |
| 2019 | Tumescence Anesthesia Solution-Assisted Laser Ablation Treatment of Lower Limb Varicose Veins: The Effect of Temperature of the Tumescence Anesthesia Solution on Intraoperative and Postoperative Pain, Clinical Observations, and Comprehensive Nursing Care. | J Perianesth Nurs | Luo, L. H., Z. Chen, L. N. Hu, C. Ma and E. H. Xiao | 10.1016/j.jopan.2018.06.091 |
| 2019 | A prospective observational cohort study of concomitant versus sequential phlebectomy for tributary varicosities following axial mechanochemical ablation. | Phlebology | Mohamed, A., C. Leung, L. Hitchman, T. Wallace, G. Smith, D. Carradice and I. Chetter | 10.1177/0268355519835625 |
| 2019 | Mechanochemical ablation for the treatment of superficial venous incompetence: A cohort study of a single centre's early experience. | Phlebology | Mohamed, A. H., C. Leung, T. Wallace, S. Pymer, A. Harwood, G. Smith, D. Carradice and I. C. Chetter | 10.1177/0268355518818339 |
| 2019 | Comparison of cyanoacrylate closure and radiofrequency ablation for the treatment of incompetent great saphenous veins: 36-Month outcomes of the VeClose randomized controlled trial. | Phlebology | Morrison, N., R. Kolluri, M. Vasquez, M. Madsen, A. Jones and K. Gibson | 10.1177/0268355518810259 |
| 2019 | Chronic venous insufficiency: a new concept to understand pathophysiology at the microvascular level - a pilot study. | Perfusion | Mutlak, O., M. Aslam and N. J. Standfield | 10.1177/0267659118791682 |
| 2019 | A Randomised Controlled Trial Comparing Three Different Radiofrequency Technologies: Short-Term Results of the 3-RF Trial. | Eur J Vasc Endovasc Surg | Nyamekye, I. K., N. Dattani, W. Hayes, D. Harding, S. Holloway and J. Newman | 10.1016/j.ejvs.2019.01.033 |
| 2019 | Comparison of ultrasound results following endovenous laser ablation and radiofrequency ablation in the treatment of varicose veins. | Ann Ital Chir | Ontas, H., T. Yavuz, A. N. Acar and D. Uysal | |
| 2019 | Significant differences in patients with a complete insufficiency of the great versus small saphenous vein. | Phlebology | Pochec, K., D. Mühlberger, T. Hummel, M. Stücker and S. Reich-Schupke | 10.1177/0268355518798277 |
| 2019 | Comparison of foam sclerotherapy versus radiofrequency ablation in the treatment of primary varicose veins due to incompetent great saphenous vein: Randomized clinical trial. | J Vasc Nurs | Rai, A., M. Porsalman, A. Khatony and M. Sobhiyeh | 10.1016/j.jvn.2019.10.002 |
| 2019 | Lower Limb Deep Vein Diameters Beneath Medical Compression Stockings in the Standing Position. | Eur J Vasc Endovasc Surg | Rastel, D. and B. Lun | 10.1016/j.ejvs.2018.07.040 |
| 2019 | Pivotal Study of Endovenous Stent Placement for Symptomatic Iliofemoral Venous Obstruction. | Circ Cardiovasc Interv | Razavi, M. K., S. Black, P. Gagne, R. Chiacchierini, P. Nicolini and W. Marston | 10.1161/circinterventions.119.008268 |
| 2019 | Patient-reported outcomes of endovenous superficial venous ablation for lower extremity swelling. | Phlebology | Shutze, W., R. Shutze, P. Dhot and G. O. Ogola | 10.1177/0268355518814130 |
| 2019 | Prospective randomized trial comparing radiofrequency ablation and complete saphenous vein stripping in patients with mild to moderate chronic venous disease with a 3-year follow-up. | Einstein (Sao Paulo) | Sincos, I. R., A. P. W. Baptista, F. Coelho Neto, N. Labropoulos, L. B. Alledi, E. M. Marins, J. Puggina, S. Q. Belczak, M. G. Cardoso and R. Aun | 10.31744/einstein_journal/2019AO4526 |
| 2019 | Institution of a Double-Dosing Protocol Using Cyanoacrylate Glue for Larger Diameter Refluxing Superficial Truncal Veins. | Dermatol Surg | Tang, T. Y., H. P. Rathnaweera, E. Choke, A. Tiwari and T. T. Chong | 10.1097/dss.0000000000001801 |
| 2019 | Randomized clinical trial of mechanochemical and endovenous thermal ablation of great saphenous varicose veins. | Br J Surg | Vähäaho, S., O. Mahmoud, K. Halmesmäki, A. Albäck, K. Noronen, P. Vikatmaa, P. Aho and M. Venermo | 10.1002/bjs.11158 |
| 2019 | Relationships between the use of pharmacomechanical catheter-directed thrombolysis, sonographic findings, and clinical outcomes in patients with acute proximal DVT: Results from the ATTRACT Multicenter Randomized Trial. | Vasc Med | Weinberg, I., S. Vedantham, A. Salter, G. Hadley, N. Al-Hammadi, C. Kearon, J. A. Julian, M. K. Razavi, H. L. Gornik, S. Z. Goldhaber, A. J. Comerota, A. L. Kindzelski, R. M. Schainfeld, J. F. Angle, S. Misra, J. A. Schor, D. Hurst and M. R. Jaff | 10.1177/1358863x19862043 |
| 2019 | Medical compression stockings on the skin moisture in patients with chronic venous disease. | Vasa | Westphal, T., W. Konschake, H. Haase, M. Vollmer, M. Jünger and H. Riebe | 10.1024/0301-1526/a000812 |
| 2019 | Positive effects of the inclusion of open-mouth pressure for elimination of blood in microscopic subinguinal varicocelectomy. | Int Urol Nephrol | Xu, C., W. Xia, Y. Sun, H. Chen and T. Song | 10.1007/s11255-019-02228-9 |
| 2019 | Comparison of cyanoacrylate embolization and radiofrequency ablation for the treatment of varicose veins. | Phlebology | Yang, G. K., M. Parapini, J. Gagnon and J. C. Chen | 10.1177/0268355518794105 |
| 2018 | Treatment of foot varicose veins: A study of 119 consecutive patients. | Phlebology | Albernaz, L. F., D. T. S. Albernaz, F. R. M. Zignani and Y. W. Chi | 10.1177/0268355517693100 |
| 2018 | Cyanoacrylate adhesive embolization and sclerotherapy for primary varicose veins. | Phlebology | Bellam Premnath, K. P., B. Joy, V. A. Raghavendra, A. Toms and T. Sleeba | 10.1177/0268355517733339 |
| 2018 | Treatment protocol on stasis edema in poorly mobile nursing home patients. | Int Angiol | Benigni, J. P., J. F. Uhl, F. Balet and M. Chahim | 10.23736/s0392-9590.18.04025-7 |
| 2018 | Prospective, double-blind, randomized controlled trial comparing electrocoagulation and radiofrequency in the treatment of patients with great saphenous vein insufficiency and lower limb varicose veins. | J Vasc Surg Venous Lymphat Disord | Beteli, C. B., F. H. Rossi, B. L. de Almeida, N. M. Izukawa, C. B. Onofre Rossi, S. A. Gabriel, A. M. Kambara, A. G. de Moraes Rego Sousa and P. Thorpe | 10.1016/j.jvsv.2017.09.010 |
| 2018 | Autologous platelet-rich plasma in the treatment of venous leg ulcers in primary care: a randomised controlled, pilot study. | J Wound Care | Burgos-Alonso, N., I. Lobato, I. Hernández, K. S. Sebastian, B. Rodríguez, A. G. March, A. Perez-Salvador, V. Arce, A. Garcia-Alvarez, M. C. Gomez-Fernandez, G. Grandes and I. Andia | 10.12968/jowc.2018.27.Sup6.S20 |
| 2018 | A potential therapeutic pitfall in the treatment of venous reflux due to variant planar anatomy of varicose segments. | Phlebology | Deniz, S., D. Tureli, B. Akpinar and L. Oguzkurt | 10.1177/0268355517722703 |
| 2018 | LivRelief varicose veins cream in the treatment of chronic venous insufficiency of the lower limbs: A 6-week single arm pilot study. | PLoS One | Dwyer, H. C., D. C. Baranowski, P. V. Mayer and S. Gabriele | 10.1371/journal.pone.0208954 |
| 2018 | A Randomised Clinical Trial Comparing N-Butyl Cyanoacrylate, Radiofrequency Ablation and Endovenous Laser Ablation for the Treatment of Superficial Venous Incompetence: Two Year Follow up Results. | Eur J Vasc Endovasc Surg | Eroglu, E. and A. Yasim | 10.1016/j.ejvs.2018.05.028 |
| 2018 | Analysis of threshold stenosis by multiplanar venogram and intravascular ultrasound examination for predicting clinical improvement after iliofemoral vein stenting in the VIDIO trial. | J Vasc Surg Venous Lymphat Disord | Gagne, P. J., A. Gasparis, S. Black, P. Thorpe, M. Passman, S. Vedantham, W. Marston and M. Iafrati | 10.1016/j.jvsv.2017.07.009 |
| 2018 | Long-term outcomes of isolated superficial vein thrombosis in patients with active cancer. | Thromb Res | Galanaud, J. P., S. Blaise, M. A. Sevestre, H. Terrisse, G. Pernod, C. Gaillard, C. Genty, M. Monreal, Y. Rabah, S. R. Kahn, I. Quéré and J. L. Bosson | 10.1016/j.thromres.2018.04.013 |
| 2018 | Twenty-four month results from a randomized trial of cyanoacrylate closure versus radiofrequency ablation for the treatment of incompetent great saphenous veins. | J Vasc Surg Venous Lymphat Disord | Gibson, K., N. Morrison, R. Kolluri, M. Vasquez, R. Weiss, D. Cher, M. Madsen and A. Jones | 10.1016/j.jvsv.2018.04.009 |
| 2018 | A Randomized Trial of Endovascular Embolization Treatment in Pelvic Congestion Syndrome: Fibered Platinum Coils versus Vascular Plugs with 1-Year Clinical Outcomes. | J Vasc Interv Radiol | Guirola, J. A., M. Sánchez-Ballestin, S. Sierre, C. Lahuerta, V. Mayoral and M. A. De Gregorio | 10.1016/j.jvir.2017.09.011 |
| 2018 | Antioxidative mechanism in the course of varicose veins. | Phlebology | Horecka, A., J. Biernacka, A. Hordyjewska, W. Dąbrowski, P. Terlecki, T. Zubilewicz, I. Musik and J. Kurzepa | 10.1177/0268355517721055 |
| 2018 | Open prospective randomized study of the results of using Venarus in postthrombotic disease. | Angiol Sosud Khir | Ignat'ev, I. M. | |
| 2018 | Phase 2 Study of an Intravenous Busulfan and Melphalan Conditioning Regimen for Autologous Stem Cell Transplantation in Patients with Multiple Myeloma (KMM150). | Biol Blood Marrow Transplant | Jung, S. H., J. J. Lee, J. S. Kim, C. K. Min, K. Kim, Y. Choi, H. S. Eom, Y. D. Joo, S. H. Kim, J. Y. Kwak, H. J. Kang, J. H. Lee, H. S. Lee, Y. C. Mun, J. H. Moon, S. K. Sohn, S. K. Park, Y. Park, H. J. Shin and S. S. Yoon | 10.1016/j.bbmt.2018.01.004 |
| 2018 | Acute Effects of Graduated Elastic Compression Stockings in Patients with Symptomatic Varicose Veins: A Randomised Double Blind Placebo Controlled Trial. | Eur J Vasc Endovasc Surg | Kakkos, S. K., M. Timpilis, P. Patrinos, K. M. Nikolakopoulos, C. P. Papageorgopoulou, A. K. Kouri, I. Ntouvas, S. I. Papadoulas, G. C. Lampropoulos and I. A. Tsolakis | 10.1016/j.ejvs.2017.10.004 |
| 2018 | Endotheliotropic effects of venotonic drugs in treatment of patients with varicose veins. | Angiol Sosud Khir | Kalinin, R. E., I. A. Suchkov, A. A. Kamaev, V. I. Zvyagina and A. A. Krylov | |
| 2018 | Symptoms Associated With Chronic Venous Disease in Response to a Cooling Treatment Compared to Placebo: A Randomized Clinical Trial. | J Wound Ostomy Continence Nurs | Kelechi, T. J., M. J. Dooley, M. Mueller, M. Madisetti and M. A. Prentice | 10.1097/won.0000000000000441 |
| 2018 | Effectiveness of cooling therapy (cryotherapy) on leg pain and self-efficacy in patients with chronic venous disease: A randomized controlled trial. | Int J Nurs Stud | Kelechi, T. J., M. Mueller, M. Madisetti, M. A. Prentice and M. J. Dooley | 10.1016/j.ijnurstu.2018.04.015 |
| 2018 | Eight-year follow-up of a randomized clinical trial comparing ultrasound-guided foam sclerotherapy with surgical stripping of the great saphenous vein. | Br J Surg | Lam, Y. L., J. A. Lawson, I. M. Toonder, N. H. Shadid, A. Sommer, M. Veenstra, A. M. J. van der Kleij, R. P. Ceulen, E. de Haan, F. Ibrahim, T. van Dooren, F. H. Nieman and C. H. A. Wittens | 10.1002/bjs.10762 |
| 2018 | Prospective comparative cohort study evaluating incompetent great saphenous vein closure using radiofrequency-powered segmental ablation or 1470-nm endovenous laser ablation with radial-tip fibers (Varico 2 study). | J Vasc Surg Venous Lymphat Disord | Lawson, J. A., S. A. Gauw, C. J. van Vlijmen, P. Pronk, M. T. W. Gaastra, M. J. Tangelder and M. C. Mooij | 10.1016/j.jvsv.2017.06.016 |
| 2018 | Prospective Comparison of Effect of Ligation and Foam Sclerotherapy with Foam Sclerotherapy Alone for Varicose Veins. | Ann Vasc Surg | Li, X., B. Yang, X. Li and S. Ren | 10.1016/j.avsg.2018.01.004 |
| 2018 | Effect of temperature for tumescence anesthesia solution on intraoperative and postoperative pain of endovenous laser ablation of lower extremity varicose vein. | Zhong Nan Da Xue Xue Bao Yi Xue Ban | Luo, L., Z. Chen, E. Xiao and C. Ma | 10.11817/j.issn.1672-7347.2018.06.012 |
| 2018 | The influence of exercise on ulcer healing in patients with chronic venous insufficiency. | Int Angiol | Mutlak, O., M. Aslam and N. Standfield | 10.23736/s0392-9590.18.03950-0 |
| 2018 | A Randomised Clinical Trial of Buffered Tumescent Local Anaesthesia During Endothermal Ablation for Superficial Venous Incompetence. | Eur J Vasc Endovasc Surg | Nandhra, S., T. Wallace, J. El-Sheikha, C. Leung, D. Carradice and I. Chetter | 10.1016/j.ejvs.2018.05.017 |
| 2018 | Treatment of incompetent perforators in recurrent venous insufficiency with adhesive embolization and sclerotherapy. | Phlebology | Prasad Bp, K., B. Joy, A. Toms and T. Sleeba | 10.1177/0268355517696612 |
| 2018 | The initial report on 1-year outcomes of the feasibility study of the VENITI VICI VENOUS STENT in symptomatic iliofemoral venous obstruction. | J Vasc Surg Venous Lymphat Disord | Razavi, M., W. Marston, S. Black, D. Bentley and P. Neglén | 10.1016/j.jvsv.2017.10.014 |
| 2018 | Advantages and disadvantages of graduated and inverse graduated compression hosiery in patients with chronic venous insufficiency and healthy volunteers: A prospective, mono-centric, blinded, open randomised, controlled and cross-over trial. | Phlebology | Riebe, H., W. Konschake, H. Haase and M. Jünger | 10.1177/0268355516682885 |
| 2018 | Randomized double-blinded study comparing medical treatment versus iliac vein stenting in chronic venous disease. | J Vasc Surg Venous Lymphat Disord | Rossi, F. H., A. M. Kambara, N. M. Izukawa, T. O. Rodrigues, C. B. Rossi, A. G. Sousa, P. B. Metzger and P. E. Thorpe | 10.1016/j.jvsv.2017.11.003 |
| 2018 | Comparative Bioavailability of Two Diosmin Formulations after Oral Administration to Healthy Volunteers. | Molecules | Russo, R., D. Chandradhara and N. De Tommasi | 10.3390/molecules23092174 |
| 2018 | Change in perception of sclerotherapy results after exposure to pre-post intervention photographs. | Phlebology | Santiago, F. R., M. Piscoya and Y. W. Chi | 10.1177/0268355517736178 |
| 2018 | Customizing elastic pressure bandages for reuse to a predetermined, sub-bandage pressure: A randomized controlled trial. | Phlebology | Sermsathanasawadi, N., T. Tarapongpun, R. Pianchareonsin, N. Puangpunngam, C. Wongwanit, K. Chinsakchai, P. Mutirangura and C. Ruangsetakit | 10.1177/0268355517746434 |
| 2018 | Individualised versus standard duration of elastic compression therapy for prevention of post-thrombotic syndrome (IDEAL DVT): a multicentre, randomised, single-blind, allocation-concealed, non-inferiority trial. | Lancet Haematol | Ten Cate-Hoek, A. J., E. E. Amin, A. C. Bouman, K. Meijer, L. W. Tick, S. Middeldorp, G. J. M. Mostard, M. Ten Wolde, S. M. van den Heiligenberg, S. van Wissen, M. H. van de Poel, S. Villalta, E. H. Serné, H. M. Otten, E. H. Klappe, I. M. Bistervels, M. N. Lauw, M. Piersma-Wichers, P. Prandoni, M. A. Joore, M. H. Prins and H. Ten Cate | 10.1016/s2352-3026(17)30227-2 |
| 2018 | Effects of intermittent pneumatic compression treatment on clinical outcomes and biochemical markers in patients at low mobility with lower limb edema. | J Vasc Surg Venous Lymphat Disord | Tessari, M., V. Tisato, E. Rimondi, P. Zamboni and A. M. Malagoni | 10.1016/j.jvsv.2018.01.019 |
| 2018 | A prolonged antibiotic protocol to treat persistent Chlamydophila pneumoniae infection improves the extracranial venous circulation in multiple sclerosis. | Phlebology | Thibault, P., J. Attia and C. Oldmeadow | 10.1177/0268355517712884 |
| 2018 | Five-year follow-up of a randomized clinical trial comparing open surgery, foam sclerotherapy and endovenous laser ablation for great saphenous varicose veins. | Br J Surg | Vähäaho, S., K. Halmesmäki, A. Albäck, E. Saarinen and M. Venermo | 10.1002/bjs.10757 |
| 2018 | Long-term outcomes of endovenous laser ablation and conventional surgery for great saphenous varicose veins. | Br J Surg | Wallace, T., J. El-Sheikha, S. Nandhra, C. Leung, A. Mohamed, A. Harwood, G. Smith, D. Carradice and I. Chetter | 10.1002/bjs.10961 |
| 2018 | Efficacy and Safety of Extracranial Vein Angioplasty in Multiple Sclerosis: A Randomized Clinical Trial. | JAMA Neurol | Zamboni, P., L. Tesio, S. Galimberti, L. Massacesi, F. Salvi, R. D'Alessandro, P. Cenni, R. Galeotti, D. Papini, R. D'Amico, S. Simi, M. G. Valsecchi and G. Filippini | 10.1001/jamaneurol.2017.3825 |
| 2018 | A 1470-nm laser combined with foam sclerotherapy in day surgery: a better choice for lower limb varicose veins. | Lasers Med Sci | Zhang, X., X. Wang, C. Gao, J. Qin, H. Zhao, W. Li and X. Lu | 10.1007/s10103-018-2507-8 |
| 2017 | Thirty-sixth-month follow-up of first-in-human use of cyanoacrylate adhesive for treatment of saphenous vein incompetence. | J Vasc Surg Venous Lymphat Disord | Almeida, J. I., J. J. Javier, E. G. Mackay, C. Bautista, D. J. Cher and T. M. Proebstle | 10.1016/j.jvsv.2017.03.016 |
| 2017 | Shortened protocol for radiofrequency ablation of perforator veins. | J Vasc Surg Venous Lymphat Disord | Aurshina, A., A. Hingorani, S. Blumberg, A. Alsheekh, N. Marks, A. Hingorani, E. Iadagarova and E. Ascher | 10.1016/j.jvsv.2017.04.010 |
| 2017 | Compression versus No Compression after Endovenous Ablation of the Great Saphenous Vein: A Randomized Controlled Trial. | Ann Vasc Surg | Ayo, D., S. N. Blumberg, C. R. Rockman, M. Sadek, N. Cayne, M. Adelman, L. Kabnick, T. Maldonado and T. Berland | 10.1016/j.avsg.2016.08.008 |
| 2017 | Prevention of thromboembolic complications in patients with superficial-vein thrombosis given rivaroxaban or fondaparinux: the open-label, randomised, non-inferiority SURPRISE phase 3b trial. | Lancet Haematol | Beyer-Westendorf, J., S. M. Schellong, H. Gerlach, E. Rabe, J. I. Weitz, K. Jersemann, K. Sahin and R. Bauersachs | 10.1016/s2352-3026(17)30014-5 |
| 2017 | Cyanoacrylate glue used to treat great saphenous reflux: Measures of outcome. | Phlebology | Chan, Y. C., Y. Law, G. C. Cheung, A. C. Ting and S. W. Cheng | 10.1177/0268355516638200 |
| 2017 | A prognostic analysis of cirrhotic esophageal variceal bleeding treated with standardized endoscopic therapy. | Zhonghua Gan Zang Bing Za Zhi | Dai, Y. P. and Q. Gao | 10.3760/cma.j.issn.1007-3418.2017.03.007 |
| 2017 | Impact of UK NICE Clinical Guidelines 168 and social deprivation on access to interventional treatment for symptomatic varicose vein and specialist referral for leg ulceration. | Phlebology | Davies, H. O., M. Popplewell, G. Bate, L. Kelly, A. Koutsoumpelis and A. W. Bradbury | 10.1177/0268355516677874 |
| 2017 | Fibrin gel versus papain gel in the healing of chronic venous ulcers: A double-blind randomized controlled trial. | Phlebology | de Araújo, I. C., E. Defune, L. P. Abbade, H. A. Miot, M. Bertanha, L. R. de Carvalho, R. R. Ferreira and W. B. Yoshida | 10.1177/0268355516664808 |
| 2017 | A multicenter, randomized, placebo-controlled study to evaluate the efficacy and safety of Varithena® (polidocanol endovenous microfoam 1%) for symptomatic, visible varicose veins with saphenofemoral junction incompetence. | Phlebology | Gibson, K. and L. Kabnick | 10.1177/0268355516635386 |
| 2017 | Sclerotherapy is a safe method of treatment of chronic venous disorders in older patients: A prospective and comparative study of consecutive patients. | Phlebology | Gillet, J. L., C. H. Desnos, M. Lausecker, C. Daniel, J. J. Guex and F. A. Allaert | 10.1177/0268355516642659 |
| 2017 | Mid-term outcome of endovascular treatment for acute lower extremity deep venous thrombosis. | Phlebology | Jiang, K., X. Q. Li, H. F. Sang, A. M. Qian, J. J. Rong and C. L. Li | 10.1177/0268355516640370 |
| 2017 | Improvement in patient-reported outcomes of varicose veins following treatment with polidocanol endovenous microfoam. | Phlebology | Lal, B. K., R. Mallick and D. Wright | 10.1177/0268355516678512 |
| 2017 | A multi-centre randomised controlled trial comparing radiofrequency and mechanical occlusion chemically assisted ablation of varicose veins - Final results of the Venefit versus Clarivein for varicose veins trial. | Phlebology | Lane, T., R. Bootun, B. Dharmarajah, C. S. Lim, M. Najem, S. Renton, K. Sritharan and A. H. Davies | 10.1177/0268355516651026 |
| 2017 | Comparison of endovenous ablation techniques, foam sclerotherapy and surgical stripping for great saphenous varicose veins. Extended 5-year follow-up of a RCT. | Int Angiol | Lawaetz, M., J. Serup, B. Lawaetz, L. Bjoern, A. Blemings, B. Eklof and L. Rasmussen | 10.23736/s0392-9590.17.03827-5 |
| 2017 | Patient-centered outcomes of a dual action pneumatic compression device in comparison to compression stockings for patients with chronic venous disease. | J Vasc Surg Venous Lymphat Disord | Lurie, F. and M. Schwartz | 10.1016/j.jvsv.2017.06.003 |
| 2017 | VeClose trial 12-month outcomes of cyanoacrylate closure versus radiofrequency ablation for incompetent great saphenous veins. | J Vasc Surg Venous Lymphat Disord | Morrison, N., K. Gibson, M. Vasquez, R. Weiss, D. Cher, M. Madsen and A. Jones | 10.1016/j.jvsv.2016.12.005 |
| 2017 | Evaluation of inflammatory cell biomarkers in chronic venous insufficiency. | Phlebology | Mosmiller, L. T., K. N. Steele, C. D. Shrader and A. B. Petrone | 10.1177/0268355517701806 |
| 2017 | Endovenous laser ablation of the great saphenous vein - Varying energy may not affect outcome. | Phlebology | Nejm, C. S., Jr., J. R. Timi, W. B. de Araújo, Jr. and F. C. Caron | 10.1177/0268355515620944 |
| 2017 | Clinical efficacy of electric stimulation of crural muscles in comprehensive treatment of post-thrombotic disease. | Angiol Sosud Khir | Ryzhkin, V. V., K. V. Lobastov, A. V. Vorontsova, I. V. Schastlivtsev, V. E. Barinov, E. K. Naumov and L. A. Laberko | |
| 2017 | Efficacy of Indocyanine Green Angiography on Microsurgical Subinguinal Varicocelectomy. | J Invest Surg | Shibata, Y., S. Kurihara, S. Arai, H. Kato, T. Suzuki, Y. Miyazawa, H. Koike, K. Ito, T. Nakamura and K. Suzuki | 10.1080/08941939.2016.1236855 |
| 2017 | Quality of life after great saphenous vein ablation in Thai patients with great saphenous vein reflux. | Asian J Surg | Siribumrungwong, B., P. Noorit, C. Wilasrusmee, Y. Teerawattananon and A. Thakkinstian | 10.1016/j.asjsur.2015.10.004 |
| 2017 | Results of administering oral anticoagulants for treatment of patients with venous thromboembolic complications. | Angiol Sosud Khir | Sukovatykh, B. S., A. V. Sereditskiĭ, V. F. Muradian, L. N. Belikov, A. M. Azarov and O. F. Gerasimova | |
| 2017 | A randomized prospective long-term (>1 year) clinical trial comparing the efficacy and safety of radiofrequency ablation to 980 nm laser ablation of the great saphenous vein. | Phlebology | Sydnor, M., J. Mavropoulos, N. Slobodnik, L. Wolfe, B. Strife and D. Komorowski | 10.1177/0268355516658592 |
| 2017 | ClariVein® - Early results from a large single-centre series of mechanochemical endovenous ablation for varicose veins. | Phlebology | Tang, T. Y., J. W. Kam and M. E. Gaunt | 10.1177/0268355516630154 |
| 2017 | Effect of Pycnogenol on the Healing of Venous Ulcers. | Ann Vasc Surg | Toledo, R. R., M. Santos and T. B. Schnaider | 10.1016/j.avsg.2016.04.014 |
| 2017 | A multicenter, randomized, placebo-controlled trial of endovenous thermal ablation with or without polidocanol endovenous microfoam treatment in patients with great saphenous vein incompetence and visible varicosities. | Phlebology | Vasquez, M. and A. P. Gasparis | 10.1177/0268355516637300 |
| 2017 | Defining the optimum tumescent anaesthesia solution in endovenous laser ablation. | Phlebology | Wallace, T., C. Leung, S. Nandhra, N. Samuel, D. Carradice and I. Chetter | 10.1177/0268355516653905 |
| 2017 | A new non-tumescent endovenous ablation method for varicose vein treatment: Early results of N-butyl cyanoacrylate (VariClose®). | Phlebology | Yasim, A., E. Eroglu, O. Bozoglan, B. Mese, M. Acipayam and H. Kara | 10.1177/0268355516638577 |
| 2017 | The efficiency of O-(beta-hydroxyethyl)-rutosides in reducing the incidence of superficial venous insufficiency in patients with calf muscle pump dysfunction. | Phlebology | Yildiz, C. E., C. Conkbayir, E. Huseynov, O. A. Sayin, O. Tok, G. Kaynak, D. Cebi, M. Ugurlucan, F. Kantarci and M. Inan | 10.1177/0268355516635466 |
| 2017 | Prospective Randomized Study of Ultrasound-Guided Foam Sclerotherapy Combined with Great Saphenous Vein High Ligation in the Treatment of Severe Lower Extremity Varicosis. | Ann Vasc Surg | Yin, H., H. He, M. Wang, Z. Li, Z. Hu, C. Yao, J. Wang, S. Wang and G. Chang | 10.1016/j.avsg.2016.06.027 |
| 2017 | The treatment of elderly and senile patients with venous trophic ulcers and type 2 diabetes mellitus.. | Adv Gerontol | Zakharova, N. O., S. V. Bulgakova, S. E. Katorkin, M. A. Melnikov, E. V. Treneva and A. V. Nikolaeva | |
| 2017 | CHIVA - A prospective study of a vein sparing technique for the management of varicose vein disease. | Am J Surg | Zmudzinski, M., P. Malo, C. Hall and A. Hayashi | 10.1016/j.amjsurg.2017.03.025 |
| 2016 | Secondary ablation of saphenous veins: The reasons and the ratios. | Phlebology | Aktas, A. R. and U. Ozkan | 10.1177/0268355515581742 |
| 2016 | Cryosclerosis. The forgotten endovenous cryoablation of the great saphenous vein. Mid-term results of a prospective comparative trial. | Orv Hetil | Bálint, I. B., Á. Farics, L. Vizsy, E. Vargovics, R. Bálint, J. Bátorfi and G. Menyhei | 10.1556/650.2016.30625 |
| 2016 | Intra-procedural pain score in a randomised controlled trial comparing mechanochemical ablation to radiofrequency ablation: The Multicentre Venefit™ versus ClariVein® for varicose veins trial. | Phlebology | Bootun, R., T. R. Lane, B. Dharmarajah, C. S. Lim, M. Najem, S. Renton, K. Sritharan and A. H. Davies | 10.1177/0268355514551085 |
| 2016 | A prospective comparison of a new cyanoacrylate glue and laser ablation for the treatment of venous insufficiency. | Phlebology | Bozkurt, A. K. and M. F. Yılmaz | 10.1177/0268355516632652 |
| 2016 | Comparison of Endovenous Laser and Radiofrequency Ablation in Treating Varicose Veins in the Same Patient. | Vasc Endovascular Surg | Bozoglan, O., B. Mese, E. Eroglu, M. B. Erdogan, K. Erdem, H. C. Ekerbicer and A. Yasim | 10.1177/1538574415625813 |
| 2016 | Effects of Wearing Compression Stockings on the Physical Performance of T2DM Men with MetS. | Int J Sports Med | Brinkmann, C., R. Hermann, E. Rühl, H. Kerzel, L. Reinhardt, M. Grau, J. Latsch, M. Kohl-Bareis, W. Bloch and K. Brixius | 10.1055/s-0035-1565202 |
| 2016 | Benefits of polidocanol endovenous microfoam (Varithena®) compared with physician-compounded foams. | Phlebology | Carugo, D., D. N. Ankrett, X. Zhao, X. Zhang, M. Hill, V. O'Byrne, J. Hoad, M. Arif, D. D. Wright and A. L. Lewis | 10.1177/0268355515589063 |
| 2016 | Behavioural recovery after treatment for varicose veins. | Br J Surg | Cotton, S. C., G. MacLennan, J. Brittenden, M. Prior and J. Francis | 10.1002/bjs.10081 |
| 2016 | The impact of 2013 UK NICE guidelines on the management of varicose veins at the Heart of England NHS Foundation Trust, Birmingham, UK. | Phlebology | Davies, H. O., M. Popplewell, G. Bate, L. Kelly, K. Darvall and A. W. Bradbury | 10.1177/0268355515610236 |
| 2016 | Endovenous laser ablation with and without high ligation compared to high ligation and stripping for treatment of great saphenous varicose veins: Results of a multicentre randomised controlled trial with up to 6 years follow-up. | Phlebology | Flessenkämper, I., M. Hartmann, K. Hartmann, D. Stenger and S. Roll | 10.1177/0268355514555547 |
| 2016 | Five-year follow-up of a randomized, controlled trial comparing saphenofemoral ligation and stripping of the great saphenous vein with endovenous laser ablation (980 nm) using local tumescent anesthesia. | J Vasc Surg | Gauw, S. A., J. A. Lawson, C. J. van Vlijmen-van Keulen, P. Pronk, M. T. Gaastra and M. C. Mooij | 10.1016/j.jvs.2015.08.084 |
| 2016 | Intralesional cryosurgery for the treatment of basal cell carcinoma of the lower extremities in elderly subjects: a feasibility study. | Int J Dermatol | Har-Shai, Y., A. Sommer, T. Gil, J. Krausz, N. Gal-Or, I. Mettanes, I. Lavi, N. Eyal, L. Brizgalin, A. Taran, L. Har-Shai and I. Elmalach | 10.1111/ijd.13168 |
| 2016 | Recurrence of superficial vein thrombosis in patients with varicose veins. | Phlebology | Karathanos, C., K. Spanos, V. Saleptsis, A. Tsezou, D. Kyriakou and A. D. Giannoukas | 10.1177/0268355515596475 |
| 2016 | Clinical acceptability study of once-daily versus twice-daily micronized purified flavonoid fraction in patients with symptomatic chronic venous disease: a randomized controlled trial. | Int Angiol | Kirienko, A. and D. Radak | |
| 2016 | Roll-in phase analysis of clinical study of cyanoacrylate closure for incompetent great saphenous veins. | J Vasc Surg Venous Lymphat Disord | Kolluri, R., K. Gibson, D. Cher, M. Madsen, R. Weiss and N. Morrison | 10.1016/j.jvsv.2016.06.017 |
| 2016 | A randomised controlled trial comparing compression therapy after radiofrequency ablation for primary great saphenous vein incompetence. | Phlebology | Krasznai, A. G., T. A. Sigterman, S. Troquay, J. P. Houtermans-Auckel, M. Snoeijs, H. G. Rensma, C. Sikkink and L. H. Bouwman | 10.1177/0268355514568658 |
| 2016 | The influence of the training of the muscular component of the musculo-venous pump in the lower extremities on the clinical course of varicose vein disease. | Vopr Kurortol Fizioter Lech Fiz Kult | Kravtsov, P. F., S. A. Katorkin, V. V. Volkovoy and Y. V. Sizonenko | 10.17116/kurort2016633-36 |
| 2016 | Clarivein® mechano-chemical ablation an interim analysis of a randomized controlled trial dose-finding study. | Phlebology | Lam, Y. L., I. M. Toonder and C. H. Wittens | 10.1177/0268355515599692 |
| 2016 | Haemodynamic Performance of Low Strength Below Knee Graduated Elastic Compression Stockings in Health, Venous Disease, and Lymphoedema. | Eur J Vasc Endovasc Surg | Lattimer, C. R., E. Kalodiki, M. Azzam and G. Geroulakos | 10.1016/j.ejvs.2016.04.001 |
| 2016 | Endogenous pro-thrombotic biomarkers from the arm and leg may not have the same value. | Phlebology | Lattimer, C. R., E. Kalodiki, G. Geroulakos, D. Hoppensteadt and J. Fareed | 10.1177/0268355515589678 |
| 2016 | Endovenous laser ablation versus mechanochemical ablation with ClariVein(®) in the management of superficial venous insufficiency (LAMA trial): study protocol for a randomised controlled trial. | Trials | Leung, C. C., D. Carradice, T. Wallace and I. C. Chetter | 10.1186/s13063-016-1548-1 |
| 2016 | Randomized clinical trial of 940- versus 1470-nm endovenous laser ablation for great saphenous vein incompetence. | Br J Surg | Malskat, W. S., J. Giang, M. G. De Maeseneer, T. E. Nijsten and R. R. van den Bos | 10.1002/bjs.10035 |
| 2016 | Randomized trial of radiofrequency ablation versus conventional surgery for superficial venous insufficiency: if you don't tell, they won't know. | Clinics (Sao Paulo) | Mendes, C. A., A. A. Martins, J. M. Fukuda, J. B. Parente, M. A. Munia, A. Fioranelli, M. P. Teivelis, A. Y. Varella, R. A. Caffaro, S. Kuzniec and N. Wolosker | 10.6061/clinics/2016(11)06 |
| 2016 | Endovenous laser ablation of the great saphenous vein comparing 1920-nm and 1470-nm diode laser. | Int Angiol | Mendes-Pinto, D., P. Bastianetto, L. Cavalcanti Braga Lyra, R. Kikuchi and L. Kabnick | |
| 2016 | Effects of Fentanyl and Morphine on Shivering During Spinal Anesthesia in Patients Undergoing Endovenous Ablation of Varicose Veins. | Med Sci Monit | Onk, D., T. Akarsu Ayazoğlu, U. Kuyrukluyıldız, M. Aksüt, Z. Bedir, İ. Küpeli, O. A. Onk and A. Alagöl | 10.12659/msm.897256 |
| 2016 | The VVSymQ® instrument: Use of a new patient-reported outcome measure for assessment of varicose vein symptoms. | Phlebology | Paty, J., D. M. Turner-Bowker, C. A. Elash and D. Wright | 10.1177/0268355515595193 |
| 2016 | A randomized, double-blind, placebo-controlled, clinical study on the efficacy and safety of calcium dobesilate in the treatment of chronic venous insufficiency. | Phlebology | Rabe, E., S. Ballarini and L. Lehr | 10.1177/0268355515586097 |
| 2016 | Efficacy of topical local anaesthesia to reduce perioperative pain for endovenous laser ablation of varicose veins: a double-blind randomized controlled trial. | Ther Adv Cardiovasc Dis | Saha, S., A. Tiwari, C. Hunns, J. Refson and A. Abidia | 10.1177/1753944716644140 |
| 2016 | Cost-utility analysis of great saphenous vein ablation with radiofrequency, foam and surgery in the emerging health-care setting of Thailand. | Phlebology | Siribumrungwong, B., P. Noorit, C. Wilasrusmee, P. Leelahavarong, A. Thakkinstian and Y. Teerawattananon | 10.1177/0268355515604258 |
| 2016 | Adherence to and efficacy of different compression methods for treating chronic venous insufficiency in the elderly. | Phlebology | Suehiro, K., N. Morikage, O. Yamashita, T. Harada, K. Ueda, M. Samura, Y. Tanaka, Y. Takeuchi and K. Hamano | 10.1177/0268355515608992 |
| 2016 | Endovenous laser ablation is an effective treatment for great saphenous vein incompetence in teenagers. | Phlebology | Terlecki, P., S. Przywara, M. Iłżecki, K. Terlecki, P. Kawecki and T. Zubilewicz | 10.1177/0268355515585436 |
| 2016 | Randomized clinical trial comparing surgery, endovenous laser ablation and ultrasound-guided foam sclerotherapy for the treatment of great saphenous varicose veins. | Br J Surg | Venermo, M., J. Saarinen, E. Eskelinen, S. Vähäaho, E. Saarinen, M. Railo, I. Uurto, J. Salenius and A. Albäck | 10.1002/bjs.10260 |
| 2016 | Primary varicose veins treated with HE's fire needle therapy: a randomized controlled trial. | Zhongguo Zhen Jiu | Wang, L., K. Chu, L. Zeng and L. Wang | |
| 2016 | Recurrence patterns after endovenous laser treatment of saphenous vein reflux. | Phlebology | Winokur, R. S., N. M. Khilnani and R. J. Min | 10.1177/0268355515596288 |
| 2016 | Post-operative Benefit of Compression Therapy after Endovenous Laser Ablation for Uncomplicated Varicose Veins: A Randomised Clinical Trial. | Eur J Vasc Endovasc Surg | Ye, K., R. Wang, J. Qin, X. Yang, M. Yin, X. Liu, M. Jiang and X. Lu | 10.1016/j.ejvs.2016.09.005 |
| 2016 | Saphenous nerve injury after endovenous laser ablation of incompetent greater saphenous vein: An electroneuromyography study. | Phlebology | Yilmaz, S., O. Delikan and E. Aksoy | 10.1177/0268355514568533 |
| 2016 | Oscillatory flow suppression improves inflammation in chronic venous disease. | J Surg Res | Zamboni, P., P. Spath, V. Tisato, M. Tessari, P. Dalla Caneva, E. Menegatti, S. Occhionorelli, S. Gianesini and P. Secchiero | 10.1016/j.jss.2016.06.046 |
| 2016 | Predicting the severity of liver cirrhosis through clinical parameters. | J Surg Res | Zhang, E. L., Z. Y. Zhang, S. P. Wang, Z. Y. Xiao, J. Gu, M. Xiong, X. P. Chen and Z. Y. Huang | 10.1016/j.jss.2016.04.036 |
| 2015 | Bemiparin for thromboprophylaxis after benign gynecologic surgery: a randomized clinical trial. | J Thromb Haemost | Alalaf, S. K., A. K. Jawad, R. K. Jawad, M. S. Ali and N. G. Al Tawil | 10.1111/jth.13164 |
| 2015 | Two-year follow-up of first human use of cyanoacrylate adhesive for treatment of saphenous vein incompetence. | Phlebology | Almeida, J. I., J. J. Javier, E. G. Mackay, C. Bautista, D. J. Cher and T. M. Proebstle | 10.1177/0268355514532455 |
| 2015 | Clinical effectiveness and cost-effectiveness of foam sclerotherapy, endovenous laser ablation and surgery for varicose veins: results from the Comparison of LAser, Surgery and foam Sclerotherapy (CLASS) randomised controlled trial. | Health Technol Assess | Brittenden, J., S. C. Cotton, A. Elders, E. Tassie, G. Scotland, C. R. Ramsay, J. Norrie, J. Burr, J. Francis, S. Wileman, B. Campbell, P. Bachoo, I. Chetter, M. Gough, J. Earnshaw, T. Lees, J. Scott, S. A. Baker, G. MacLennan, M. Prior, D. Bolsover and M. K. Campbell | 10.3310/hta19270 |
| 2015 | A prospective randomized study comparing polidocanol foam sclerotherapy with surgical treatment of patients with primary chronic venous insufficiency and ulcer. | Ann Vasc Surg | Campos, W., Jr., I. O. Torres, E. S. da Silva, I. B. Casella and P. Puech-Leão | 10.1016/j.avsg.2015.01.031 |
| 2015 | Low-grade elastic compression regimen for venous leg ulcers--an effective compromise for patients requiring daily dressing changes. | Int Wound J | Dabiri, G., S. Hammerman, P. Carson and V. Falanga | 10.1111/iwj.12186 |
| 2015 | Maggots as a wound debridement agent for chronic venous leg ulcers under graduated compression bandages: A randomised controlled trial. | Phlebology | Davies, C. E., G. Woolfrey, N. Hogg, J. Dyer, A. Cooper, J. Waldron, R. Bulbulia, M. R. Whyman and K. R. Poskitt | 10.1177/0268355514555386 |
| 2015 | Influence of manual lymphatic drainage on health-related quality of life and symptoms of chronic venous insufficiency: a randomized controlled trial. | Arch Phys Med Rehabil | dos Santos Crisóstomo, R. S., D. S. Costa, C. de Luz Belo Martins, T. I. Fernandes and P. A. Armada-da-Silva | 10.1016/j.apmr.2014.09.020 |
| 2015 | Ovarian Vein Diameter Cannot Be Used as an Indicator of Ovarian Venous Reflux. | Eur J Vasc Endovasc Surg | Dos Santos, S. J., J. M. Holdstock, C. C. Harrison, A. J. Lopez and M. S. Whiteley | 10.1016/j.ejvs.2014.10.013 |
| 2015 | Two-year results of a prospective randomised controlled multicenter trial to compare open operative therapy vs. endoluminal venous laser therapy with and without high ligation for the therapy of varicose greater saphenous veins. | Zentralbl Chir | Flessenkämper, I. H., D. Stenger, M. Hartmann, K. Hartmann and S. Roll | 10.1055/s-0033-1360347 |
| 2015 | Less painful tumescent solution for patients undergoing endovenous laser ablation of the saphenous vein. | Ann Vasc Surg | Gunes, T., F. Altin, B. Kutas, S. Aydin, K. Erkoc, B. Eygi, I. Alur and F. Ozdemir | 10.1016/j.avsg.2015.02.010 |
| 2015 | Thermal ablation of saphenous veins is feasible and safe in patients older than 75 years: A prospective study (EVTA study). | Phlebology | Hamel-Desnos, C., P. Desnos, F. A. Allaert and P. Kern | 10.1177/0268355514540882 |
| 2015 | The impact of hand reflexology on pain, anxiety and satisfaction during minimally invasive surgery under local anaesthetic: a randomised controlled trial. | Int J Nurs Stud | Hudson, B. F., J. Davidson and M. S. Whiteley | 10.1016/j.ijnurstu.2015.07.009 |
| 2015 | Randomized controlled trial to compare the effect of simple distraction interventions on pain and anxiety experienced during conscious surgery. | Eur J Pain | Hudson, B. F., J. Ogden and M. S. Whiteley | 10.1002/ejp.675 |
| 2015 | A thematic analysis of experiences of varicose veins and minimally invasive surgery under local anaesthesia. | J Clin Nurs | Hudson, B. F., J. Ogden and M. S. Whiteley | 10.1111/jocn.12719 |
| 2015 | Saphenous vein stripping surgical technique and frequency of saphenous nerve injury. | Phlebology | Jaworucka-Kaczorowska, A., G. Oszkinis, J. Huber, A. Wiertel-Krawczuk, E. Gabor and P. Kaczorowski | 10.1177/0268355514539316 |
| 2015 | Impact of graduated compression stockings on the prevention of post-thrombotic syndrome - results of a randomized controlled trial. | Phlebology | Jayaraj, A. and M. Meissner | 10.1177/0268355514544781 |
| 2015 | Five-year results of a randomized controlled trial comparing high ligation combined with endovenous laser ablation and stripping of the great saphenous vein. | Dermatol Surg | Kalteis, M., P. Adelsgruber, S. Messie-Werndl, O. Gangl and I. Berger | 10.1097/dss.0000000000000369 |
| 2015 | Histological difference between pulsed wave laser and continuous wave laser in endovenous laser ablation. | Phlebology | Kansaku, R., N. Sakakibara, A. Amano, H. Endo, T. Shimabukuro and M. Sueishi | 10.1177/0268355514538248 |
| 2015 | Treatment of Truncal Incompetence and Varicose Veins with a Single Administration of a New Polidocanol Endovenous Microfoam Preparation Improves Symptoms and Appearance. | Eur J Vasc Endovasc Surg | King, J. T., M. O'Byrne, M. Vasquez and D. Wright | 10.1016/j.ejvs.2015.06.111 |
| 2015 | Prospective study of a single treatment strategy for local tumescent anesthesia in Muller phlebectomy. | Ann Vasc Surg | Krasznai, A. G., T. A. Sigterman, C. E. Willems, P. Dekkers, M. G. Snoeijs, C. H. Wittens, C. J. Sikkink and L. H. Bouwman | 10.1016/j.avsg.2014.10.028 |
| 2015 | Does the direction of tumescent solution delivery matter in endovenous laser ablation of the great saphenous vein? | Ther Adv Cardiovasc Dis | Kutas, B., F. Ozdemir, O. Tezcan, T. Gunes, K. Erkoc, F. Altin and O. Karahan | 10.1177/1753944715599729 |
| 2015 | Ambulatory varicosity avulsion later or synchronized (AVULS): a randomized clinical trial. | Ann Surg | Lane, T. R., D. Kelleher, A. C. Shepherd, I. J. Franklin and A. H. Davies | 10.1097/sla.0000000000000790 |
| 2015 | A pilot randomized controlled trial comparing CABG surgery performed with total arterial grafts or without. | J Cardiothorac Surg | Le, J., R. J. Baskett, K. J. Buth, G. M. Hirsch, A. Brydie, R. Gayner and J. F. Legare | 10.1186/s13019-014-0203-8 |
| 2015 | A Comparison of 1,470-nm Endovenous Laser Ablation and Radiofrequency Ablation in the Treatment of Great Saphenous Veins 10 mm or More in Size. | Ann Vasc Surg | Mese, B., O. Bozoglan, E. Eroglu, K. Erdem, M. Acipayam, H. C. Ekerbicer and A. Yasim | 10.1016/j.avsg.2015.03.063 |
| 2015 | Randomized trial comparing cyanoacrylate embolization and radiofrequency ablation for incompetent great saphenous veins (VeClose). | J Vasc Surg | Morrison, N., K. Gibson, S. McEnroe, M. Goldman, T. King, R. Weiss, D. Cher and A. Jones | 10.1016/j.jvs.2014.11.071 |
| 2015 | A randomized clinical trial of endovenous laser ablation versus conventional surgery for small saphenous varicose veins. | J Vasc Surg | Nandhra, S., J. El-sheikha, D. Carradice, T. Wallace, P. Souroullas, N. Samuel, G. Smith and I. C. Chetter | 10.1016/j.jvs.2014.09.037 |
| 2015 | Treatment of severe chronic venous insufficiency with ultrasound-guided foam sclerotherapy: a two-year series in a single center in Brazil. | Phlebology | Neto, F. C., G. R. de Araújo, I. M. Kessler, R. F. de Amorim and D. P. Falcão | 10.1177/0268355513517225 |
| 2015 | Effect of Micronized Purified Flavonoid Fraction Therapy on Endothelin-1 and TNF-α Levels in Relation to Antioxidant Enzyme Balance in the Peripheral Blood of Women with Varicose Veins. | Curr Vasc Pharmacol | Pietrzycka, A., M. Kózka, T. Urbanek, M. Stpniewski and M. Kucharzewski | 10.2174/1570161113666150827124714 |
| 2015 | Analysis of the effects of micronized purified flavonoid fraction versus placebo on symptoms and quality of life in patients suffering from chronic venous disease: from a prospective randomized trial. | Int Angiol | Rabe, E., G. B. Agus and K. Roztocil | |
| 2015 | A Randomized Prospective Study Comparing Outcomes of Angioplasty versus VIABAHN Stent-Graft Placement for Cephalic Arch Stenosis in Dysfunctional Hemodialysis Accesses. | J Vasc Interv Radiol | Rajan, D. K. and A. Falk | 10.1016/j.jvir.2015.05.001 |
| 2015 | Same Site Recurrence is More Frequent After Endovenous Laser Ablation Compared with High Ligation and Stripping of the Great Saphenous Vein: 5 year Results of a Randomized Clinical Trial (RELACS Study). | Eur J Vasc Endovasc Surg | Rass, K., N. Frings, P. Glowacki, S. Gräber, W. Tilgen and T. Vogt | 10.1016/j.ejvs.2015.07.020 |
| 2015 | Interface pressure and venous drainage of two compression stocking types in healthy volunteers and in patients with hemodynamic disturbances of the legs. | Clin Hemorheol Microcirc | Riebe, H., W. Konschake, H. Haase and M. Jünger | 10.3233/ch-151989 |
| 2015 | Venous Hemodynamic Insufficiency Severity Score variation after endovascular treatment of chronic cerebrospinal venous insufficiency. | Phlebology | Scalise, F., E. Novelli, M. Farina, L. Barbato and S. Spagnolo | 10.1177/0268355514524193 |
| 2015 | Predictors of recurrence of great saphenous vein reflux following treatment with ultrasound-guided foamsclerotherapy. | Phlebology | Shadid, N., P. Nelemans, J. Lawson and A. Sommer | 10.1177/0268355514521183 |
| 2015 | COST-EFFECTIVENESS OF RADIOFREQUENCY ABLATION VERSUS LASER FOR VARICOSE VEINS. | Int J Technol Assess Health Care | Shepherd, A. C., M. Ortega-Ortega, M. S. Gohel, D. Epstein, L. C. Brown and A. H. Davies | 10.1017/s0266462315000537 |
| 2015 | Donning devices (foot slips and frames) enable elderly people with severe chronic venous insufficiency to put on compression stockings. | Eur J Vasc Endovasc Surg | Sippel, K., B. Seifert and J. Hafner | 10.1016/j.ejvs.2014.11.005 |
| 2015 | Acoustic reflectors are visible in the right heart during radiofrequency ablation of varicose veins. | Phlebology | Sounderajah, V., H. M. Moore, A. Thapar, T. R. Lane, K. Fox, I. J. Franklin and A. H. Davies | 10.1177/0268355514542680 |
| 2015 | Endovenous ablation of incompetent truncal veins and their perforators with a new radiofrequency system. Mid-term outcomes. | Vascular | Spiliopoulos, S., V. Theodosiadou, A. Sotiriadi and D. Karnabatidis | 10.1177/1708538114564462 |
| 2015 | Endovenous laser ablation in patients with wide diameter of the proximal segment of the great saphenous vein: Comparison of methods. | Phlebology | Starodubtsev, V., M. Lukyanenko, A. Karpenko and P. Ignatenko | 10.1177/0268355514555546 |
| 2015 | Radiofrequency ablation of the great saphenous vein, comparing one versus two treatment cycles for the proximal vein segment. | Phlebology | Sufian, S., A. Arnez, N. Labropoulos, K. Nguyen, V. Satwah, J. Marquez, A. Chowla and S. Lakhanpal | 10.1177/0268355514556142 |
| 2015 | Durability of treatment effect with polidocanol endovenous microfoam on varicose vein symptoms and appearance (VANISH-2). | J Vasc Surg Venous Lymphat Disord | Todd, K. L., 3rd and D. I. Wright | 10.1016/j.jvsv.2015.03.003 |
| 2015 | Compression versus inner sole for venous patients with foot static disorders: a prospective trial comparing symptoms and quality of life. | Phlebology | Uhl, J. F., M. Chahim and F. A. Allaert | 10.1177/0268355513505508 |
| 2015 | Five-year results of a randomized clinical trial of conventional surgery, endovenous laser ablation and ultrasound-guided foam sclerotherapy in patients with great saphenous varicose veins. | Br J Surg | van der Velden, S. K., A. A. Biemans, M. G. De Maeseneer, M. A. Kockaert, P. W. Cuypers, L. M. Hollestein, H. A. Neumann, T. Nijsten and R. R. van den Bos | 10.1002/bjs.9867 |
| 2015 | Conservative versus surgical treatment of venous leg ulcers: 10-year follow up of a randomized, multicenter trial. | Phlebology | van Gent, W. B., F. S. Catarinella, Y. L. Lam, F. H. Nieman, I. M. Toonder, A. C. van der Ham and C. H. Wittens | 10.1177/0268355514568848 |
| 2015 | Endovenous laser ablation of varicose veins with the 1470 nm diode laser using a radial fiber - 1-year follow-up. | Phlebology | von Hodenberg, E., C. Zerweck, M. Knittel, T. Zeller and T. Schwarz | 10.1177/0268355513512825 |
| 2015 | Lower pain and faster treatment with mechanico-chemical endovenous ablation using ClariVein®. | Phlebology | Vun, S. V., S. T. Rashid, N. C. Blest and J. I. Spark | 10.1177/0268355514553693 |
| 2015 | Postoperative prophylaxis of venous thromboembolism (VTE) in patients undergoing high ligation and stripping of the great saphenous vein (GSV). | Vasc Med | Wang, H., Z. Sun, W. Jiang, Y. Zhang, X. Li and Y. Wu | 10.1177/1358863x14564592 |
| 2014 | A randomized controlled trial of a mixed Kinesio taping-compression technique on venous symptoms, pain, peripheral venous flow, clinical severity and overall health status in postmenopausal women with chronic venous insufficiency. | Clin Rehabil | Aguilar-Ferrándiz, M. E., A. M. Castro-Sánchez, G. A. Matarán-Peñarrocha, R. Guisado-Barrilao, M. C. García-Ríos and C. Moreno-Lorenzo | 10.1177/0269215512469120 |
| 2014 | Effect of a mixed kinesio taping-compression technique on quality of life and clinical and gait parameters in postmenopausal women with chronic venous insufficiency: double-blinded, randomized controlled trial. | Arch Phys Med Rehabil | Aguilar-Ferrándiz, M. E., C. Moreno-Lorenzo, G. A. Matarán-Peñarrocha, F. García-Muro, M. C. García-Ríos and A. M. Castro-Sánchez | 10.1016/j.apmr.2014.03.024 |
| 2014 | Veno-active drugs for chronic venous disease: A randomized, double-blind, placebo-controlled parallel-design trial. | Phlebology | Belczak, S. Q., I. R. Sincos, W. Campos, J. Beserra, G. Nering and R. Aun | 10.1177/0268355513489550 |
| 2014 | Polidocanol versus hypertonic glucose for sclerotherapy treatment of reticular veins of the lower limbs: study protocol for a randomized controlled trial. | Trials | Bertanha, M., M. L. Sobreira, C. E. Pinheiro Lúcio Filho, J. V. de Oliveira Mariúba, R. E. Farres Pimenta, R. G. Jaldin, A. Moroz, R. Moura, H. A. Rollo and W. B. Yoshida | 10.1186/1745-6215-15-497 |
| 2014 | Mechanochemical ablation in patients with chronic venous disease: a prospective multicenter report. | Phlebology | Bishawi, M., R. Bernstein, M. Boter, D. Draughn, C. F. Gould, C. Hamilton and J. Koziarski | 10.1177/0268355513495830 |
| 2014 | Mechanochemical endovenous ablation versus radiofrequency ablation in the treatment of primary small saphenous vein insufficiency (MESSI trial): study protocol for a randomized controlled trial. | Trials | Boersma, D., R. R. van Eekeren, H. J. Kelder, D. A. Werson, S. Holewijn, M. A. Schreve, M. M. Reijnen and J. P. de Vries | 10.1186/1745-6215-15-421 |
| 2014 | A randomized trial comparing treatments for varicose veins. | N Engl J Med | Brittenden, J., S. C. Cotton, A. Elders, C. R. Ramsay, J. Norrie, J. Burr, B. Campbell, P. Bachoo, I. Chetter, M. Gough, J. Earnshaw, T. Lees, J. Scott, S. A. Baker, J. Francis, E. Tassie, G. Scotland, S. Wileman and M. K. Campbell | 10.1056/NEJMoa1400781 |
| 2014 | A multicenter randomized controlled trial evaluating balneotherapy in patients with advanced chronic venous insufficiency. | J Vasc Surg | Carpentier, P. H., S. Blaise, B. Satger, C. Genty, C. Rolland, C. Roques and J. L. Bosson | 10.1016/j.jvs.2013.08.002 |
| 2014 | A comparison of the effectiveness of treating those with and without the complications of superficial venous insufficiency. | Ann Surg | Carradice, D., T. Wallace, R. Gohil and I. Chetter | 10.1097/sla.0000000000000541 |
| 2014 | Clinical risk factors to predict deep venous thrombosis post-endovenous laser ablation of saphenous veins. | Phlebology | Chi, Y. W. and T. C. Woods | 10.1177/0268355512474254 |
| 2014 | Risk factors for recurrent events in subjects with superficial vein thrombosis in the randomized clinical trial SteFlux (Superficial Thromboembolism Fluxum). | Thromb Res | Cosmi, B., M. Filippini, F. Campana, G. Avruscio, A. Ghirarduzzi, E. Bucherini, G. Camporese, D. Imberti, C. Legnani and G. Palareti | 10.1016/j.thromres.2013.12.005 |
| 2014 | Catheter-directed foam sclerotherapy of great saphenous veins in combination with pre-treatment reduction of the diameter employing the principals of perivenous tumescent local anesthesia. | Eur J Vasc Endovasc Surg | Devereux, N., A. L. Recke, L. Westermann, A. Recke and B. Kahle | 10.1016/j.ejvs.2013.10.017 |
| 2014 | Manual lymphatic drainage in chronic venous disease: a duplex ultrasound study. | Phlebology | Dos Santos Crisóstomo, R. S., M. S. Candeias, A. M. Ribeiro, C. da Luz Belo Martins and P. A. Armada-da-Silva | 10.1177/0268355513502787 |
| 2014 | Haemostatic activation and inflammatory response after three methods of treatment of great saphenous vein incompetence. | Phlebology | Dzieciuchowicz, L., G. Espinosa and J. A. Páramo | 10.1177/0268355512474445 |
| 2014 | Clinical outcomes and quality of life 5 years after a randomized trial of concomitant or sequential phlebectomy following endovenous laser ablation for varicose veins. | Br J Surg | El-Sheikha, J., S. Nandhra, D. Carradice, T. Wallace, N. Samuel, G. E. Smith and I. C. Chetter | 10.1002/bjs.9565 |
| 2014 | Use of Flavonoids for the treatment of symptoms after hemorrhoidectomy with radiofrequency scalpel. | Eur Rev Med Pharmacol Sci | Filingeri, V., O. Buonomo and D. Sforza | |
| 2014 | Balneohydrotherapy in the treatment of chronic venous insufficiency. | Vasa | Forestier, R. J., G. Briancon, A. Francon, F. B. Erol and J. M. Mollard | 10.1024/0301-1526/a000374 |
| 2014 | Is the treatment of the small saphenous veins with foam sclerotherapy at risk of deep vein thrombosis? | Phlebology | Gillet, J. L., M. Lausecker, M. Sica, J. M. Guedes and F. A. Allaert | 10.1177/0268355513497362 |
| 2014 | Endovenous laser ablation and sclerotherapy for incompetent vein of Giacomini. | Phlebology | Guzelmansur, I., L. Oguzkurt, N. Koca, C. Andic, M. Gedikoglu and U. Ozkan | 10.1177/0268355513496552 |
| 2014 | Efficacy of a short course of complex lymphedema therapy or graduated compression stocking therapy in the treatment of post-thrombotic syndrome. | Vasc Med | Holmes, C. E., N. M. Bambace, P. Lewis, P. W. Callas and M. Cushman | 10.1177/1358863x14521883 |
| 2014 | Impact of compression stockings on calf-vein diameters and on quality of life parameters in subjects with painful legs. | Vasa | Jeanneret, C., K. Karatolios and I. von Planta | 10.1024/0301-1526/a000362 |
| 2014 | Selective retention of the great saphenous vein to prevent saphenous nerve injury during varicose vein surgery. | Eur Rev Med Pharmacol Sci | Jia, G. L., H. L. Xi, X. K. Wang, S. Feng and Z. L. Tian | |
| 2014 | The use of a novel method of endovenous steam ablation in treatment of great saphenous vein insufficiency: own experiences. | Phlebology | Mlosek, R. K., W. Woźniak, L. Gruszecki and R. Z. Stapa | 10.1258/phleb.2012.012092 |
| 2014 | 1064 nm Nd:YAG long pulse laser after polidocanol microfoam injection dramatically improves the result of leg vein treatment: a randomized controlled trial on 517 legs with a three-year follow-up. | Phlebology | Moreno-Moraga, J., A. Smarandache, M. L. Pascu, J. Royo and M. A. Trelles | 10.1177/0268355513502786 |
| 2014 | Pulmonary gas exchange after foam sclerotherapy. | JAMA Dermatol | Moro, L., I. Rossi Bartoli, M. Cesari, S. Scarlata, F. M. Serino and R. Antonelli Incalzi | 10.1001/jamadermatol.2013.6092 |
| 2014 | Dilution of a mepivacaine-adrenaline solution in isotonic sodium bicarbonate for reducing subcutaneous infiltration pain in ambulatory phlebectomy procedures: a randomized, double-blind, controlled trial. | J Am Acad Dermatol | Moro, L., F. M. Serino, S. Ricci, G. Abbruzzese and R. Antonelli-Incalzi | 10.1016/j.jaad.2014.06.018 |
| 2014 | Endovenous laser ablation of the great saphenous vein versus high ligation: long-term results. | Lasers Med Sci | Mozafar, M., K. Atqiaee, H. Haghighatkhah, M. S. Taheri, A. Tabatabaey and S. Lotfollahzadeh | 10.1007/s10103-013-1389-z |
| 2014 | The impact of direction of great saphenous vein total stripping on saphenous nerve injury. | Phlebology | Papakostas, J. C., E. Douitsis, I. Sarmas, S. Avgos, A. Kyritsis and M. Matsagkas | 10.1258/phleb.2012.012061 |
| 2014 | ABOUT PATIENTS, INVENTORS", JOURNALISTS, SCIENTISTS AND IRBs (TO SAY NOTHING OF THE INSTITUTIONS): CCSVI AND MS." | Med Law | Piga, M. A. | |
| 2014 | Percutaneous angioplasty of internal jugular and azygous veins in patients with chronic cerebrospinal venous insufficiency and multiple sclerosis: early and mid-term results. | Phlebology | Radak, D., J. Kolar, D. Sagic, N. Ilijevski, S. Tanaskovic, N. Aleksic, J. Marinkovic, A. Mitrasinovic, S. Radak, S. Babic, P. Matic and H. Vlajinac | 10.1177/0268355513481766 |
| 2014 | Efficacy and comfort of medical compression stockings with low and moderate pressure six weeks after vein surgery. | Phlebology | Reich-Schupke, S., F. Feldhaus, P. Altmeyer, A. Mumme and M. Stücker | 10.1177/0268355513484142 |
| 2014 | Efficacy and safety of autologous platelet rich plasma for the treatment of vascular ulcers in primary care: Phase III study. | BMC Fam Pract | San Sebastian, K. M., I. Lobato, I. Hernández, N. Burgos-Alonso, M. C. Gomez-Fernandez, J. L. López, B. Rodríguez, A. G. March, G. Grandes and I. Andia | 10.1186/s12875-014-0211-8 |
| 2014 | Compression therapy versus surgery in the treatment of patients with varicose veins: A RCT. | Eur J Vasc Endovasc Surg | Sell, H., P. Vikatmaa, A. Albäck, M. Lepäntalo, A. Malmivaara, O. Mahmoud and M. Venermo | 10.1016/j.ejvs.2014.02.015 |
| 2014 | Balloon assisted valsalva maneuver in the diagnosis of saphenofemoral junction incompetence. | J Med Assoc Thai | Sermsathanasawadi, N., K. Pattarakittikul, K. Hongku, C. Wongwanit, C. Ruangsetakit, K. Chinsakchai and P. Mutirangura | |
| 2014 | Analysis of efficacy of radiofrequency obliteration with due regard for the target vein's diameter. | Angiol Sosud Khir | Shaĭdakov, E. V., A. G. Grigorian, E. A. Iliukhin, V. L. Bulatov and M. I. Gal'chenko | |
| 2014 | Prospective randomized trial of venous angioplasty in MS (PREMiSe). | Neurology | Siddiqui, A. H., R. Zivadinov, R. H. Benedict, Y. Karmon, J. Yu, M. L. Hartney, K. L. Marr, V. Valnarov, C. L. Kennedy, M. Ramanathan, D. P. Ramasamy, K. Dolic, D. W. Hojnacki, E. Carl, E. I. Levy, L. N. Hopkins and B. Weinstock-Guttman | 10.1212/wnl.0000000000000638 |
| 2014 | Effect of graduated compression stockings on venous blood velocity in supine resting hospitalized patients. | Clin Appl Thromb Hemost | Stein, P. D., F. Matta, M. W. Akkad, C. L. Hoppe, Y. R. Patel and S. Sivakumar | 10.1177/1076029613479821 |
| 2014 | Cost-effectiveness of ultrasound-guided foam sclerotherapy, endovenous laser ablation or surgery as treatment for primary varicose veins from the randomized CLASS trial. | Br J Surg | Tassie, E., G. Scotland, J. Brittenden, S. C. Cotton, A. Elders, M. K. Campbell, B. Campbell, M. Gough, J. M. Burr and C. R. Ramsay | 10.1002/bjs.9595 |
| 2014 | The VANISH-2 study: a randomized, blinded, multicenter study to evaluate the efficacy and safety of polidocanol endovenous microfoam 0.5% and 1.0% compared with placebo for the treatment of saphenofemoral junction incompetence. | Phlebology | Todd, K. L., 3rd and D. I. Wright | 10.1177/0268355513497709 |
| 2014 | Randomized clinical trial of endovenous laser ablation versus steam ablation (LAST trial) for great saphenous varicose veins. | Br J Surg | van den Bos, R. R., W. S. Malskat, M. G. De Maeseneer, K. P. de Roos, D. A. Groeneweg, M. A. Kockaert, H. A. Neumann and T. Nijsten | 10.1002/bjs.9580 |
| 2014 | Mechanochemical endovenous Ablation versus RADiOfrequeNcy Ablation in the treatment of primary great saphenous vein incompetence (MARADONA): study protocol for a randomized controlled trial. | Trials | van Eekeren, R. R., D. Boersma, S. Holewijn, A. Vahl, J. P. de Vries, C. J. Zeebregts and M. M. Reijnen | 10.1186/1745-6215-15-121 |
| 2014 | One-shot scleroembolization: a new technique for the treatment of varicose veins disease of lower extremities. Preliminary results. | Phlebology | Viani, M. P., G. M. Viani and J. Sergenti | 10.1177/0268355513499556 |
| 2014 | Can deep vein thrombosis be predicted after varicose vein operation in women in rural areas? | Ann Agric Environ Med | Warot, M., T. Synowiec, A. Wencel-Warot, P. Daroszewski, I. Bojar, M. Micker and P. Chęciński | 10.5604/12321966.1120610 |
| 2014 | Catheter-directed foam sclerotherapy for chronic venous leg ulcers. | Phlebology | Williamsson, C., P. Danielsson and L. Smith | 10.1177/0268355513505506 |
